# The Hippo Pathway Transducers YAP1/TEAD Induce Acquired Resistance to Trastuzumab in HER2-Positive Breast Cancer

**DOI:** 10.3390/cancers12051108

**Published:** 2020-04-29

**Authors:** Paula González-Alonso, Sandra Zazo, Ester Martín-Aparicio, Melani Luque, Cristina Chamizo, Marta Sanz-Álvarez, Pablo Minguez, Gonzalo Gómez-López, Ion Cristóbal, Cristina Caramés, Jesús García-Foncillas, Pilar Eroles, Ana Lluch, Oriol Arpí, Ana Rovira, Joan Albanell, Sander R. Piersma, Connie R. Jimenez, Juan Madoz-Gúrpide, Federico Rojo

**Affiliations:** 1 Pathology, Fundación Jiménez Díaz University Hospital Health Research Institute (IIS—FJD, UAM)—CIBERONC, 28040 Madrid, Spain; 2 Genetics Department, Health Research Institute-Fundación Jiménez Díaz (IIS-FJD, UAM), Center for Biomedical Network Research on Rare Diseases (CIBERER), ISCIII, 28040 Madrid, Spain; 3 Bioinformatics Unit, Spanish National Cancer Research Centre (CNIO), 28029 Madrid, Spain; 4 Translational Oncology Division, OncoHealth Institute, Health Research Institute-Fundación Jiménez Díaz (IIS-FJD, UAM), 28040 Madrid, Spain; 5 Institute of Health Research INCLIVA-CIBERONC, 46010 Valencia, Spain; 6 Cancer Research Program, IMIM (Hospital del Mar Research Institute), 08003 Barcelona, Spain; 7 Medical Oncology Department, Hospital del Mar-CIBERONC, 08003 Barcelona, Spain; 8 CEXS Department, Pompeu Fabra University, 08002 Barcelona, Spain; 9 OncoProteomics Laboratory, Department of Medical Oncology, Cancer Center Amsterdam, Amsterdam University Medical Center (location VUmc), 1081 HV Amsterdam, The Netherlands

**Keywords:** breast cancer, anti-receptor therapy, trastuzumab, resistance, Hippo pathway, YAP1, TEAD

## Abstract

Trastuzumab is the first-line targeted therapeutic drug for HER2-positive breast cancer, leading to improved overall survival. However, acquired resistance inevitably occurs. We aimed to identify, quantify, and assess the mechanisms of acquired resistance to trastuzumab. We established an acquired trastuzumab-resistant model in vitro from BT-474, a trastuzumab-sensitive, HER2-amplified breast-cancer cell line. A multi-omic strategy was implemented to obtain gene, proteome, and phosphoproteome signatures associated with acquired resistance to trastuzumab in HER2-positive breast cancer, followed by validation in human clinical samples. YAP1 dephosphorylation and *TEAD2* overexpression were detected as significant alterations in the Hippo pathway in trastuzumab-resistant breast cancer. Because of the emerging role of these proteins as mediators of normal growth and tumorigenesis, we assessed the exogenous modulation of their activity, either by in vitro gene silencing or by pharmacological inhibition of the YAP1/TEAD complexes, both in vitro and in vivo. Moreover, we identified increased signaling through the Hippo pathway in human samples after progression following trastuzumab treatment. Finally, YAP1/TAZ nuclear accumulation in malignant cells in HER2 breast tumor was significantly associated with worse progression-free and overall survival in metastatic HER2-positive breast-cancer patients. Our results suggest the involvement of Hippo signaling in acquired trastuzumab resistance in breast cancer. Additionally, we provide novel evidence for a potential breast-cancer treatment strategy based on dual targeting of HER2 and Hippo pathway effectors, which may improve the antitumor activity of trastuzumab and help overcome resistance.

## 1. Introduction

Among the molecular markers employed in routine clinical practice for prognosis and predictive aims, human epidermal growth factor receptor 2 (HER2; ErbB2) defines a discrete category of breast tumors with specific characteristics. The gene is amplified in 20–25% of human breast cancer patients, leading to HER2 protein overexpression and resulting in constitutive HER2 tyrosine kinase activation and downstream signaling [1]. Amplification of HER2 in breast cancer patients correlates with disease progression, poor survival outcome, and disease recurrence [2]. Importantly, the development of the monoclonal anti-HER2 antibody trastuzumab has been shown to have a substantial clinical benefit, inhibiting tumor progression by targeting the HER2 receptor in early and metastatic breast-cancer patients [3,4]. Despite this effectiveness, in early stage settings, over 20% of HER2-positive breast cancer patients are insensitive to trastuzumab treatment due to de novo resistance, and in metastatic settings most HER2-positive breast-cancer patients may develop resistance to trastuzumab after 6 to 8 months of exposure to the treatment and suffer disease progression within one year [4]. Thus, there is a pressing need to further our knowledge of the molecular bases that limit the response to trastuzumab in order to discover predictive biomarkers, overcome resistance to standard anti-HER2 treatment by developing alternative therapies, and eventually enhance treatment response and survival for HER2-positive breast-cancer patients [3,5].

Control of cell proliferation, cell differentiation, and apoptosis are crucial biological processes in human development and tissue homeostasis, and as a result the deregulation of these processes may lead to organ degeneration or tumor formation. Included among the emerging genes regulating these processes is the highly conserved Hippo signaling pathway, which controls cell proliferation, apoptosis, and stemness, and consequently regulates organ size, tissue growth, and homeostasis [6,7]. Deregulation of the Hippo tumor-suppressor pathway was observed in human cancer, including lung, colorectal, ovarian, liver, and breast cancer (reviewed in [6]). Yes-associated protein (YAP1) and transcriptional co-activator with PDZ-binding motif (TAZ) can be considered the key effectors of the Hippo signaling cascade. When the Hippo pathway is inactive, YAP1/TAZ effectors are dephosphorylated and translocate to the nucleus, where they mediate major physiological functions of the Hippo pathway by regulating gene transcription [8]. The transcriptional enhanced associated domain (TEAD) family of transcription factors comprises the major mediators of YAP1/TAZ in the Hippo pathway, and these factors are critical for YAP1 regulation of cell proliferation, cell death, and oncogenic transformation [9,10].

Emerging evidence suggests that the Hippo pathway could modulate both primary and acquired resistance to anti-cancer therapies. Recent studies show that YAP1 promotes resistance to epidermal growth factor receptor (EGFR) tyrosine kinase inhibitors (TKIs) in lung cancer [11] or inhibitors of the Raf–mitogen-activated protein kinase (MAPK)/extracellular signal-regulated kinase (ERK) kinase MEK–ERK–MAPK signaling pathway in BRAF- and RAS-mutant human melanoma patients [12]. Similarly, a deregulated Hippo pathway induces chemoresistance in esophageal cancer [13] and breast cancer [14]. Of interest, findings from recent studies indicate that YAP1/TEAD interaction may be a potential therapeutic target [15,16]. In particular, an FDA-approved porphyrin compound named verteporfin has been identified as a strong inhibitor of YAP1/TEAD interaction [15]. 

In the present study, we aim to identify, quantify, and functionally evaluate potential biomarkers involved in acquired resistance to trastuzumab in an in vivo breast-cancer model [17] by means of a multi-omic strategy. In light of data pointing to a role played by YAP1/TAZ, TEAD1, and TEAD2 as mediators of normal growth and tumorigenesis [18], we then assessed the exogenous modulation of their activity, either by gene silencing or by pharmacological inhibition with verteporfin, both in vitro and in vivo. Notably, our results reveal the unexpected involvement of these proteins in the development of trastuzumab resistance in HER2-positive breast cancer, the first such finding to date. Furthermore, given the clinical benefit of targeting the Hippo pathway effectors in cancer treatment [19], we provide novel insights for a potential breast-cancer treatment strategy based on dual targeting of the HER2 receptor and Hippo pathway effectors, which may improve the antitumor activity of trastuzumab and help overcome trastuzumab resistance.

## 2. Results

### 2.1. Acquisition of Trastuzumab Resistance Confirmed Increased Signaling from HER Family Members and Epithelial-Mesenchymal Transition

**Figure 1:**
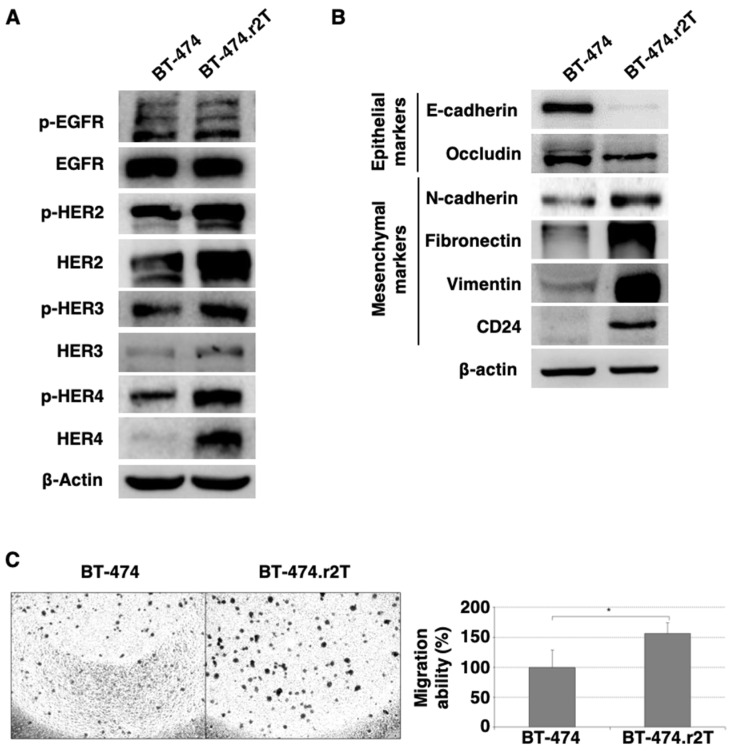
(**A**) The generation of trastuzumab resistance induced changes in the abundance and phosphorylation patterns of HER family members in BT-474.r2T cell line. Trastuzumab-sensitive and -resistant BT-474 cells were compared by WB for their protein levels at basal no-treatment conditions. β-actin was used as a loading control. (**B**) Changes were observed for different markers of EMT. (**C**) The acquisition of resistance to trastuzumab enhances transwell migration in resistant cells as compared to parental, sensitive BT-474 cells (* denotes *p* < 0.05).

The study of the status of the HER family of receptors (both total and phosphorylated forms) suggested that, in the case of the BT-474.r2T line, mechanisms other than signaling by any HER-receptor family members should be involved: WB analyses revealed an increase in abundance levels for HER2, HER3, and HER4 proteins, as well as their respective phosphorylated forms, as compared to the parental, trastuzumab-sensitive BT-474 cell line (Figure 1A). At the same time, we verified a switch in the phenotype to epithelial-mesenchymal transition (EMT), which may be indicative of a more aggressive behavior, in accordance with the other cellular findings associated with the acquired resistance (Figure 1B): we observed, in resistant BT-474.r2T cells, a downregulation of epithelial markers (E-cadherin, occludin), upregulation of mesenchymal markers (N-cadherin, fibronectin, vimentin), as well as an increase in the level of CD24. Additionally, we observed significantly increased migration in BT-474.r2T cells in comparison with BT-474 control cells (Figure 1C), thereby evidencing that resistance acquisition plays a relevant role in regulating the migration of the cells.

### 2.2. Phosphoproteome Analysis Reveals Changes in Post-Translational Regulation in Trastuzumab-Resistant Cells

A multi-omic strategy was used to identify genes, proteins, and pathways involved in acquired resistance to trastuzumab. Protein expression, phosphorylation and differential mRNA expression analyses were performed for the parental and trastuzumab-resistant cell line. For a comprehensive understanding of the molecular mechanisms involved in acquired resistance to trastuzumab in HER2-positive breast cancer, a global phosphoproteome analysis based on discovery SILAC approach for was performed in 12 samples: lysates from BT-474 and BT-474.r2T cells, which were either untreated or exposed to 15 µg/mL trastuzumab, and collected at three different times: 12 min, to capture rapid changes in phosphorylation patterns; 24 h, to identify sustained variations related to the acquisition of resistance; and 7 days, to measure the response of the cells to treatment with trastuzumab. This led us to perform a pooled analysis of the 12 samples to extend the statistical power of the study. The results show a comparative analysis of 12 experiments: 2 untreated controls (light-labeled BT-474 and heavy-labeled BT-474.r2T, baseline conditions) and 2 treated conditions (heavy-labeled BT-474 and light-labeled BT-474.r2T, exposed to trastuzumab) each one for either 12 min, 24 h, or 7 days. This strategy allowed us to identify resistance-related, trastuzumab-independent regulation of phosphorylation events within BT-474 and BT-474.r2T cells. On average, Class 1 phosphosites were 74% of all phosphosites identified for each assay (Table S1), and the overall results were similar in each time condition, revealing a reproducible phosphopeptide enrichment rate within samples. These phosphosites were derived from 89,744 unique peptides, including 1679 different phosphoprotein groups (mean values).

Our analytical strategy focused on changes in resistance and non-response regulation, initially based on phosphopeptide alterations, to reveal markers of acquired mechanisms. To that end, we combined four conditions: either (i) differential regulation in resistant vs. parental cells, or (ii) differential regulation between treated resistant and sensitive cells, plus (iii) non-regulation in resistant cells after trastuzumab treatment or regulation in the same direction, or (iv) regulation relevant for sensitivity (in opposite direction as in previous conditions) in short term trastuzumab treatment (“de novo regulation”). The whole analytical strategy can be summarized in mathematical terms as follows: [(BT-474.r2T vs. BT-474) ∪ (BT-474.r2T + T vs. BT-474 + T)] ∩ (BT-474.r2T + T vs. BT-474.r2T)\(BT-474 + T vs. BT-474).

**Figure 2:**
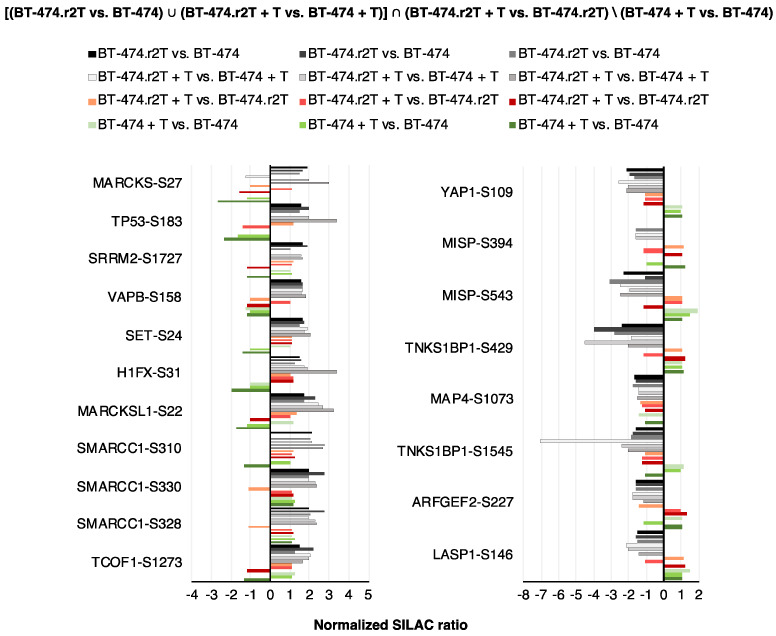
Phosphoproteomics analysis identified up- and downregulated candidates in acquired trastuzumab-resistant BT-474.r2T cells compared to parental sensitive BT-474 cells. Proteins selected in the phosphoproteomic SILAC assay according to their significantly downregulated or overexpressed phosphosites in BT-474.r2T vs. BT-474 cells (overlapping of the three datasets). Bar graphs represent FC of Class-1 phosphosites identified in different temporary conditions, with annotation of the modified residue.

We initially considered an overlapping result of at least 2 out of 3 of the datasets (12 min, 24 h, 7 days), which unveiled 43 downregulated class-1 phosphosites (Figure S1A) and 43 up-regulated (Figure S1B) class-1 phosphosites, corresponding to 46 phosphopeptides, for a cutoff SILAC ratio of 1.5 in all the 12 experiments. Among the downregulated phosphosites in resistant cells. The overlapping of the 3 datasets (12 min, 24 h, 7 days) increased stringency to expose proteins commonly altered in all conditions, which finally resulted in the identification of 8 downregulated class-1 phosphosites (Figure S1A) and 11 upregulated class-1 phosphosites (Figure S1B), corresponding to 15 phosphopeptides in BT-474.r2T cells in a trastuzumab-independent manner. YAP1 showed the most consistent pattern of “acquired resistance marker plus surrogate marker for trastuzumab non-efficiency”. In particular, we detected decreased phosphorylation of YAP1-Ser109 in BT-474.r2T cells for every condition measuring resistance, with no variation due to trastuzumab treatment, and small reverse modulation in BT-474 cells (Figure 2).

### 2.3. Gene Expression Analysis Highlights Changes in Transcriptional Expression Levels of TEAD2 Associated with Acquisition of Trastuzumab Resistance

For a comprehensive understanding of the genes involved in acquired resistance to trastuzumab in HER2-positive breast cancer, we analyzed the gene expression profile of BT-474 and BT-474.r2T cells using Affymetrix microarrays. A PCA revealed a clear separation between BT-474 and BT-474.r2T cells (Figure S2). Genes whose expression levels were significantly different (FDR-adjusted *p* < 0.05 and FC > 2) between trastuzumab-resistant and sensitive cells were plotted on a heatmap, which revealed that 36 genes showed trastuzumab-independent differential regulation of gene expression in BT-474 and BT-474.r2T cells (16 genes were significantly upregulated in BT-474.r2T cells, whereas 20 genes were significantly downregulated; Figure S3).

Among the top genes differentially expressed between sensitive and resistant cells, we identified a group of candidates related to tumor-microenvironment response (CCL5, CXCL10, IFIT3, IFI44, IFNL2). This profile confirmed our previous results about the involvement of C-C motif chemokine ligand 5 (CCL5)/C-C motif chemokine receptor 5 (CCR5) in acquired resistance to trastuzumab in HER2-positive breast cancer (article in press). Other than that, differential expression analysis of BT-474.r2T vs. BT-474 cells (at baseline and upon trastuzumab treatment) revealed *Tead2* overexpression in BT-474.r2T cells, with a 2.24-fold increase (FDR-adjusted *p* < 0.05). Based on its negative regulation of cell death, its emerging oncogenic role in mediating transcription of genes involved in cancer-cell proliferation, cell migration, and angiogenesis [10], as well as its key role in the Hippo signaling pathway, TEAD2 was selected for further studies of molecular mechanisms of resistance to trastuzumab.

Additionally, an integrated, pathway-focused insight on the resistance-specific differences in gene expression was also elucidated by GSEA analysis. The resistant phenotype was characterized at baseline condition by increased expression of ErbB2/HER2, ER, EGFR, and MEK signatures (Table S2A); similarly, at treatment condition, resistant cells showed decreased expression of ErbB2/HER2, ER, and MEK signatures, while signatures of enriched expression of fibroblast predicting cancer progression were included (Table S2B).

### 2.4. Multi-Omic Strategy Reveals Changes in YAP1 and TEAD2 Regulation in Trastuzumab-Resistant Cells

**Figure 3:**
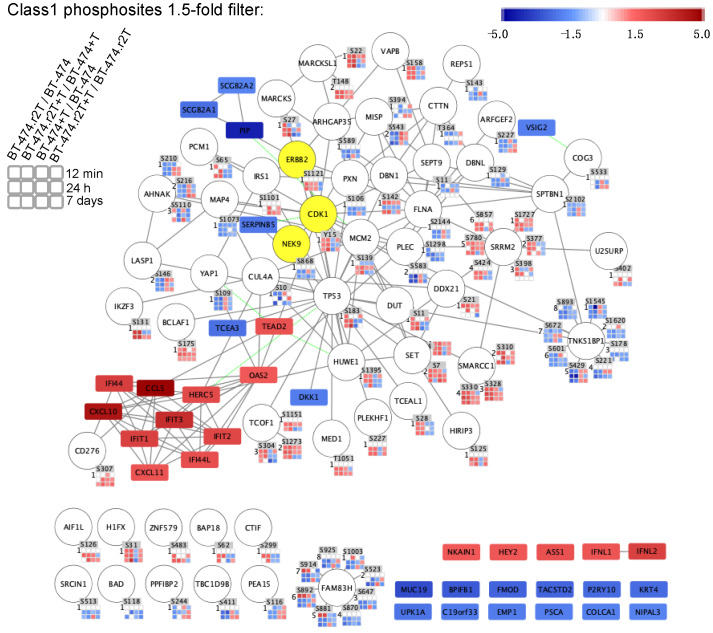
Multi-omic study identified up- and downregulated candidates in acquired trastuzumab-resistant BT-474.r2T cells compared to parental sensitive BT-474 cells. Prediction map of molecular interactions among candidates involved in our model of acquired resistance to trastuzumab, according to phosphorylation signals of the identified candidates by SILAC phosphoproteomic analysis (≥two out of three overlaps, ≥1.5-fold change) and mRNA expression microarrays (BT-474 vs. BT-474.r2T cells, both untreated and treated with trastuzumab, ≥ 2-fold change). Ellipse nodes shows identified proteins. Phosphorylated residues are indicated, and squares show quantification time points for SILAC experiments; rectangle shows identified mRNAs. Negative regulation (blue scale: 43 Class 1 phosphosites, 19 mRNAs) and positive regulation (red scale: 43 Class 1 phosphosites, 16 mRNAs) are indicated; yellow-labeled nodes show the known kinases identified; white-labelled time points shows missing values.

As both SILAC and gene microarrays constitute discovery marker approaches, we integrated both sets of proteome and genome expression data into a comprehensive, two-level network to gain further detail into the mechanism of resistance acquisition. Differentially expressed genes (BT-474 vs. BT-474.r2T cells, both untreated and treated with trastuzumab, ≥ 2-fold change) and phosphosites (overlap of 2 out of 3 datasets ≥ 1.5-fold change) with information from described quantitative time series were visualized in Phosphopath, showing significant deregulation of YAP1 and TEAD2 in trastuzumab-resistant cells compared to parental cells (Figure 3). This extended network showed upregulation of kinases ERBB2 and (cyclin dependent kinase 1) CDK1, while NIMA related kinase 9 (NEK9) was downregulated in the BT-474.r2T cell line (Figure 3). Proteins involved in cancer, such as thyroid hormone receptor associated protein 3 (BCLAF2), tumor protein p53 (TP53), mediator complex subunit 1 (MED1), and others confirmed that the signaling was that of a tumor cell. Visualization of more stringent filtering (containing overlap within three SILAC datasets) showed downregulation of YAP1-Ser109 phosphorylation and TEAD2 overexpression, connected to the overexpressed cytokines, such as CCL5, identified and previously reported as resistance drivers in additional models by our group (Figure S4).

GO enrichment analysis of transcriptome and phosphoproteome data (overlapping of the three datasets) highlighted the enrichment in biological processes of identified candidates in response to cytokine-mediated signaling pathways, type-I interferon signaling pathway, immune response, and epithelium development, among others. Consideration of less restrictive conditions revealed other biological processes, and these were reduced into less redundant terms as response to virus, regulation of cell differentiation, and macromolecular complex subunit organization (BH FDR corrected *p* ≤ 0.05; Table S3).

### 2.5. YAP1/TEAD2 Transcriptional Activity Upregulation in HER2-Positive Breast Cancer Cells Induced by Acquired Trastuzumab Resistance

**Figure 4:**
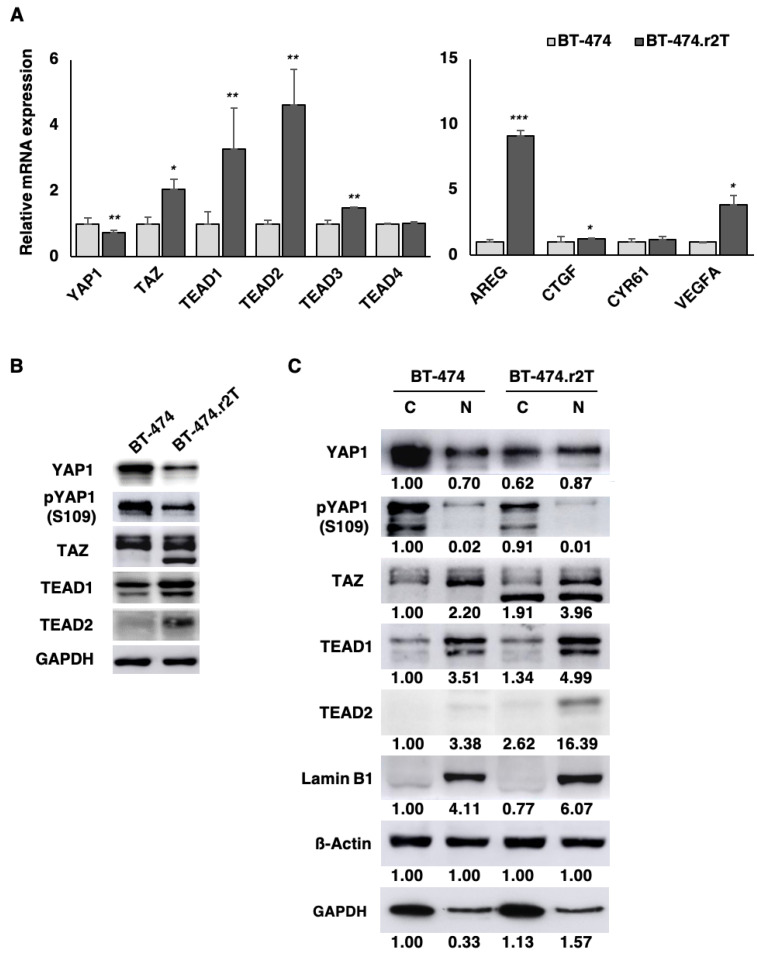
TEAD1 and TEAD2 are highly expressed in trastuzumab-resistant BT-474.r2T cells and, together with YAP1, are upregulated in response to acquired trastuzumab resistance. 2.5E6 cells were seeded in 6-multiwell plates, and after 96 h of treatment, whole cell protein isolation, nuclear-cytoplasm protein fractionation, or RNA isolation were performed. (**A**) qPCR analysis of relative *YAP1*, *TAZ*, *TEAD1*, *TEAD2*, *TEAD3*, *TEAD4*, *AREG*, *CTGF*, *CYR61*, and *VEGFA* mRNA expression following in vitro generation of trastuzumab-resistant BT-474.r2T cells (*: *p* < 0.05, **: *p* < 0.01, ***: *p* < 0.001). (**B**) WB analysis of total YAP1, pYAP1-Ser109, TAZ, TEAD1, and TEAD2 expression in cells shown in (**A**). (**C**) WB analysis of total YAP, pYAP-Ser109, TAZ, TEAD1 and TEAD2 expression in the nuclear/cytosol extracts of cells shown in (**A**). Relative abundance levels of protein up- or downregulation were determined by densitometric analysis of the images.

Among the candidates associated with acquisition of resistance, YAP1 and TEAD2 were mainly included in processes of regulation of cell differentiation, as well as epithelium development. Given that dysregulation of the pathways controlled by YAP1 and TEAD family members exerts a significant impact on cancer development and therapeutic resistance [7,18], we decided to explore the impact of our findings in the generation of resistance to trastuzumab in human breast cancer. Thus, we examined the expression levels of the pivotal effectors of the Hippo pathway, finding that the transcriptional coactivators YAP1 and TAZ, as well as the TEAD family of transcription factors were validated using qPCR and immunoblotting assays, in both trastuzumab-sensitive BT-474 and trastuzumab-resistant BT-474.r2T cells. Gene-expression data revealed significantly reduced *YAP1* mRNA levels (*p* = 0.008; Figure 4A) as well as increased *TAZ* mRNA levels (*p* = 0.04) in BT-474.r2T cells. Additionally, the analysis confirmed not only overexpression of *TEAD2* (*p* = 0.005) but also upregulation of *TEAD1* (*p* = 0.04), and nearly undetectable upregulation of *TEAD3* (*p* = 0.03). *TEAD4* expression was, conversely, unmodified (*p* = 0.17) in BT-474.r2T cells. Moreover, resistant cells expressed a decreased inactivated form of pYAP1 levels compared to parental BT-474 cells (ratio = 0.60, average densitometric analysis) (Figure 4B). We also confirmed that TEAD2 abundance increased in BT-474.r2T whole-cell lysates, as compared to parental BT-474 cells (Figure 4B). Of interest, YAP1 transcriptional activity can be inferred by its subcellular localization. According to this, subcellular fractionation studies revealed predominantly nuclear YAP1 localization in BT-474.r2T cells (ratio nucleus/cytoplasm = 1.40, average densitometric analysis) as compared to BT-474 cells (n/c ratio = 0.70) (Figure 4C). Moreover, the phosphorylation level inside the nucleus decreased in resistant BT-474.r2T down to 23% (with respect to the total content of pYAP1-Ser109 in the cell). Taken together, the translocation into the nucleus and the decrease in phosphorylation level, these results are indicative of an activation of YAP1. However, this was not the case for TAZ, as results suggested that cellular localization of TAZ did not change from cytoplasm to nucleus in the resistant cells (n/c ratio = 2.20 in BT-474; n/c ratio = 2.07 in BT-474.r2T; Figure 4C). These data confirmed the findings from the SILAC approach with respect to YAP1 (Figure 2) and the gene expression analysis for TEAD2 (Figure S3) and are also in accordance with the results previously seen for YAP1, TAZ, TEAD1, and TEAD2 by qPCR (Figure 4A). Altogether, these findings suggest that YAP1—but not TAZ—transcriptional activity increased upon acquisition of resistance to trastuzumab in BT-474.r2T cells. 

To unravel the pathway downstream, we also assessed the expression levels of a set of downstream target effectors of YAP1 involved in cell proliferation, cell death, and angiogenesis (including amphiregulin [AREG], connective tissue growth factor [CTGF], cysteine-rich angiogenic inducer 61 [CYR61], and vascular endothelial growth factor A [VEGFA]). Relative transcriptional analyses showed significant overexpression of *AREG* (*p* = 4 × 10^−5^), *CTGF* (*p* = 0.02), and *VEGFA* (*p* = 0.03), while *CYR61* expression was unmodified in BT-474.r2T cells (Figure 4A). All these data suggested that increased activation of YAP1/TEAD in HER2-positive cells might directly affect the transcription of a set of genes related to cell proliferation and cell death. The altered regulation of these genes could be closely associated with the acquisition of resistance to trastuzumab in our model, and eventually with cancer progression.

### 2.6. YAP1, TEAD1, and TEAD2 Activation Drives Acquired Trastuzumab Resistance in BT-474.r2T Cells

**Figure 5:**
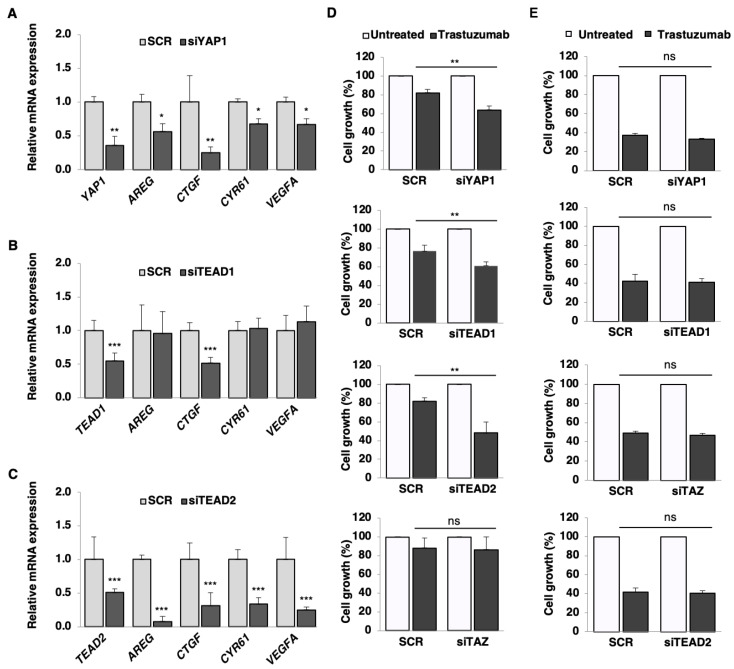
YAP1, TEAD1, and TEAD2 regulate the response to anti-HER2 inhibitor trastuzumab in BT-474.r2T cells by downregulation of specific downstream targets. (**A**) mRNA relative expression levels of *YAP1* and its targets *AREG*, *CTGF*, *CYR61,* and *VEGFA* after YAP1 silencing in the BT-474.r2T cell line. (**B**) Quantitative analyses of mRNA relative expression levels of *TEAD1* and YAP1-TEAD targets after TEAD1 silencing. (**C**) qPCR analysis of *TEAD2* mRNA expression levels and YAP1-TEAD targets after TEAD2 silencing. (**D**) Validation of the effects of either YAP1, TEAD1, TEAD2, or TAZ knockdown (siRNA) on sensitivity to trastuzumab in the trastuzumab-resistant BT-474.r2T cell line. Cell proliferation was measured after 7 days of exposure to 15 µg/mL trastuzumab. Assessment of cell growth by trypan blue exclusion cell viability assays is shown, with quantification for each condition relative to cells expressing the scrambled control siRNA. (**E**) Validation of the effects of YAP1, TEAD1, TEAD2, and TAZ knockdown on sensitivity to trastuzumab in the trastuzumab-sensitive BT-474 cell line. In all graphs, *: *p* < 0.05, **: *p* < 0.01, ***: *p* < 0.001.

As anti-HER2 directed monotherapy has incomplete efficacy in HER2-positive trastuzumab-resistant tumors, we tested whether exogenous silencing of either YAP1, TEAD1, or TEAD2 enhanced the response to anti-HER2 treatment in BT-474.r2T cells. Immunoblotting analysis confirmed decreased expression of the proteins in both cytoplasmic and nuclear fractions of RNA-silenced BT-474.r2T cells (Figure S5). qPCR revealed siYAP1 downregulation (*p* = 0.001), and consistently, YAP1 knockdown lead to a downregulation of the target genes *AREG* (*p* = 0.03), *CTGF* (*p* = 0.001), *CYR61* (*p* = 0.03), and *VEGFA* (*p* = 0.03) expression (Figure 5A). Compared to the absence of effect seen in untreated siRNA-transfected cells (80% cell growth) in BT-474.r2T cells, silencing showed enhanced sensitivity to trastuzumab (45–65% cell growth for the three different siRNAs; *p* < 0.005) in this model (Figure 5D). Thus, transient YAP1 silencing confirmed our initial transcriptome and phosphoproteome screening results and suggested a role of YAP1 in trastuzumab resistance (*p* = 0.005, Figure 5D). Moreover, a similar effect on sensitivity to trastuzumab was observed after silencing of TEAD1 in BT-474.r2T cells (*p* = 4.7 × 10^−4^; Figure 5B). While significant downregulation of *CTGF* (*p* = 2.1 × 10^−4^) was observed after TEAD1 depletion, expression of *AREG*, *CYR61*, and *VEGFA* was not affected (Figure 5B). Further exposure to 7 days of trastuzumab treatment after siTEAD1 silencing resulted in 60% cell growth, compared to SCR-transfected BT-474.r2T cells (*p* = 0.007; Figure 5D). Furthermore, significant downregulation of TEAD2 mRNA expression (*p* = 8.1 × 10^−4^, Figure 5C) and protein expression (Figure S5) was reached by siRNA silencing strategy. Consistent with these findings, TEAD2 knockdown led to a decrease in the expression of *AREG* (*p* = 2.9 × 10^−7^), *CTGF* (*p* = 0.002), *CYR61* (*p* = 3.2 × 10^−6^), and *VEGFA* (*p* = 1.3 × 10^−8^) (Figure 5C). Transient silencing of TEAD2 also restored trastuzumab sensitivity in BT-474.r2T cells (40% cell growth, *p* = 0.006, Figure 5D). Finally, to discard the involvement of TAZ in the mechanism of generation of resistance, we attempted to modulate the response of the resistant cell line to trastuzumab under TAZ-silencing conditions. In contrast with siYAP1, no effect was observed when siTAZ-cells were treated with 15 µg/mL trastuzumab (*p* > 0.05, Figure 5D). These data indicated that reduced expression of either YAP1, TEAD1, or TEAD2 restored sensitivity of HER2-positive trastuzumab-resistant BT-474.r2T cells to trastuzumab-induced antitumor effects. Moreover, we found that the YAP1/TEAD1-2–mediated transcription of *AREG*, *CTGF*, *CYR61*, and *VEGFA* might be relevant for regulation of the response to trastuzumab. Control experiments were performed in the trastuzumab-sensitive BT-474 cell line, and no significative effects due to silencing were found (Figure 5E).

### 2.7. Verteporfin-Mediated YAP1/TEAD1-2 Blockade Reduces Proliferation in HER2-Positive Breast Cancer Trastuzumab-Resistant Cells

To assess whether the inhibitor of YAP1/TEAD interaction verteporfin can affect HER2-positive cancer-cell growth and proliferation, we performed a pharmacological assay to evaluate its effects on both sensitive and trastuzumab-resistant cell lines (Figure S6). Furthermore, its combined effect with trastuzumab was assessed; thus, cells were either left untreated (DMSO control), treated with 15 µg/mL trastuzumab, treated with 50 nM verteporfin, or treated with trastuzumab and verteporfin combination for 7 days. Drug synergy studies were performed to stablish the working concentrations of the dual trastuzumab plus verteporfin treatment (Figure S7). Next, the action of verteporfin was evaluated in addition to the silencing of Hippo pathway components: proliferation studies performed in the trastuzumab-sensitive BT-474 cell line showed that, even when YAP/TAZ or TEAD1/2 were downregulated by siRNA silencing, the presence of verteporfin did not add an extra effect (Figure S8).

**Figure 6:**
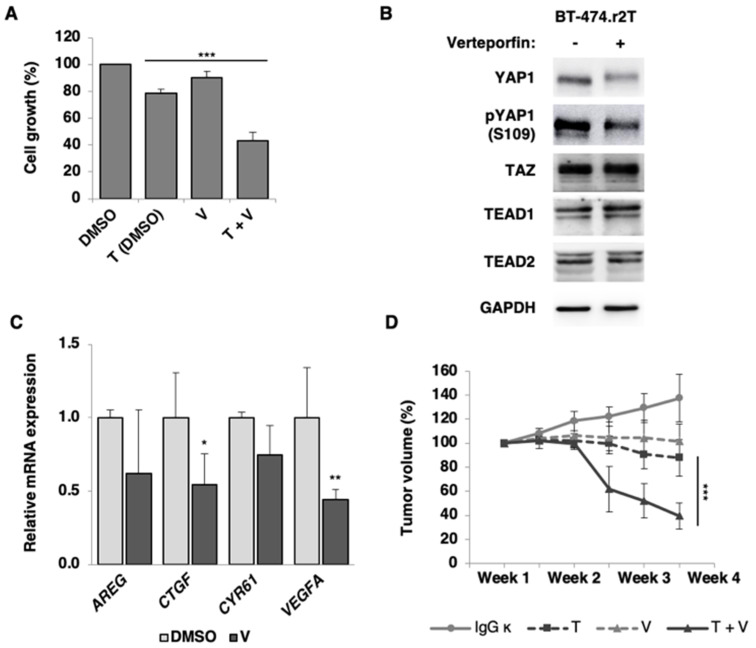
Verteporfin-mediated regulation of the YAP1/TEAD signaling pathway restores sensitivity to antitumor activity of trastuzumab in BT-474.r2T cell line. (**A**) For in vitro experiments, 2.5 × 10^6^ BT-474.r2T cells were seeded in 6-multiwell plates, and treatments were initiated after 96 h. For cell proliferation assays, BT-474.r2T cells left untreated (DMSO control), treated with 15 µg/mL trastuzumab, with 50 nM verteporfin, or with a combination of trastuzumab and verteporfin for 7 days. (**B**) WB of YAP1, pYAP1, TEAD1, and TEAD2 proteins were extracted after 48 h of 5 µM verteporfin treatment. Data are representative out of at least three independent replicates. (**C**) For the mRNA expression analyses of *AREG*, *CTGF*, *CYR61*, and *VEGFA*, cells were left untreated (DMSO control) or treated with 5 µM verteporfin for 2 h. (**D**) Combination of trastuzumab and verteporfin decreases tumor proliferation and enhances apoptosis in mouse BT-474.r2T xenografts resistant to trastuzumab. Tumor growth and statistical analysis of control (10 mg/kg IgG ĸ), 10 mg/kg trastuzumab, 40 mg/kg with verteporfin, and combined trastuzumab with verteporfin groups of treatment.

On the contrary, in the resistant cell model, exposure to combined treatment with trastuzumab and verteporfin resulted in a significant decrease of BT-474.r2T proliferation rate (45%), compared to treatment with trastuzumab alone (80%; *p* = 4.9 × 10^−4^; Figure 6A). At the molecular level, verteporfin decreased YAP1 and pYAP1 levels (Figure 6B). This reduction in dephosphorylation levels anticipated a diminished activation of the YAP1/TEAD1-2 transcription factor and subsequent underexpression of its targets, as it was later confirmed: a significant downregulation of *CTGF* (*p* = 0.02) and *VEGFA* (*p* = 0.002) gene expression was revealed, plus a slight downregulation of *AREG* and *CYR61* expression, after exposure to trastuzumab in combination with verteporfin in trastuzumab-resistant BT-474.r2T cells compared to untreated cells (Figure 6C).

### 2.8. Verteporfin-Mediated YAP1/TEAD1-2 Blockade Inhibits Tumor Resistance to Trastuzumab In Vivo

BT-474.r2T cells were xenografted in a heterotopic mouse model to further examine the role of YAP1 and TEAD1-2 in tumor growth to evaluate in vivo the potential antitumor activity of verteporfin alone or in combination with trastuzumab. Compared with mice treated with control IgG ĸ or with either trastuzumab or verteporfin treatments alone (140%, 100%, and 90%, respectively), mice treated with the combination of trastuzumab and verteporfin developed significantly reduced tumor growth (40%; *p* = 4 × 10^−4^; Figure 6D). Interestingly, verteporfin significantly enhanced trastuzumab-induced antitumor effects, confirming our previous observations in vitro. Thus, having observed that increased YAP1/TEAD activity influenced BT-474.r2T cell proliferation, we aimed to assess the effects of the combined treatment of trastuzumab and verteporfin over the proliferation and apoptosis of tumor cells by measuring the expression of pH3 and c-casp3 in mouse BT-474.r2T xenografts. Tumor specimens were collected at the end of the experiments, and the drug-mediated effects on tumor-cell proliferation and induction of apoptosis markers were further analyzed by IHC (Figure S9A). We observed that combined treatment of trastuzumab with verteporfin significantly reduced pH3 expression (*p* = 1.2 × 10^−4^, Figure S9B) and increased c-casp3 expression (*p* = 7.2 × 10^−5^, Figure S9C) in vivo, compared to single treatment with trastuzumab, which correlated with a significant decrease in tumor proliferation and enhancement of apoptosis rates, respectively. Thus, in our tumor xenograft model established from trastuzumab-resistant BT-474.r2T cells, HER2-directed therapy with trastuzumab in combination with verteporfin led to a significant inhibition of tumor growth and cell proliferation, and stimulation of apoptosis. Taken together, these results therefore suggested that YAP1/TEAD1-2 can affect tumor growth in HER2-positive breast cancer, likely by promoting cell proliferation.

### 2.9. Overexpression of Hippo Effectors Associated with Lower OS in Patients with HER2-Positive Breast Cancer 

We analyzed mRNA expression levels of the effectors of the Hippo pathway (YAP1, TAZ, TEADs) in a series of 1097 HER2-positive breast-cancer patients from available data on TCGA and correlated with OS. A tendency toward association of *YAP1* overexpression with worse OS was observed, although this was non-significant (*p* = 0.073). Additionally, an association of *TAZ* (*p* = 0.005) and *TEAD1* (*p* = 0.001) overexpression with worse OS was demonstrated (Figure S10).

### 2.10. Clinical Significance and Impact on Trastuzumab Resistance of Nuclear YAP1 Expression in Metastatic HER2 Breast-Cancer Patients

**Figure 7:**
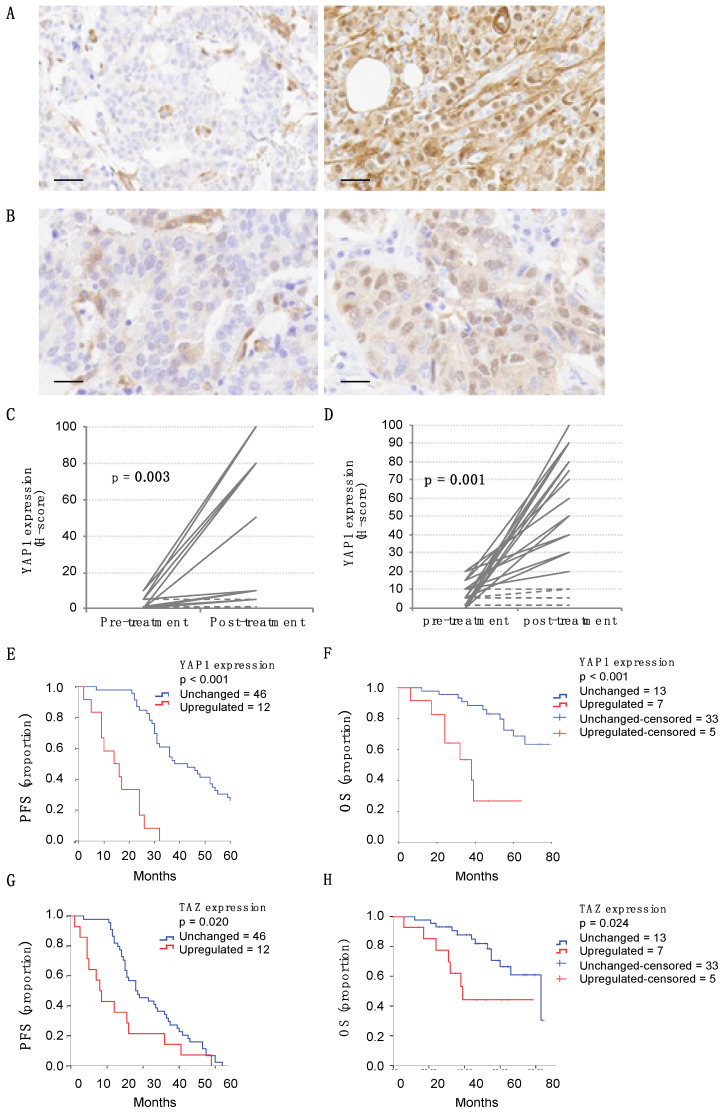
YAP1 expression impacts clinical significance in metastatic HER2 breast cancer patients and on trastuzumab resistance. (**A**) Representative IHC images of YAP1 staining from tumoral samples from two different breast cancer patients. Bar: 50 µm. (**B**) Representative IHC images of YAP1 staining from a paired sample, before and after treatment with trastuzumab (pre- and post-therapy). The percentage of tumor cells positively stained for YAP1 was estimated in 10% and 80%, respectively. Bar: 50 µm. (**C**) YAP1 expression levels in pre- and post-treatment settings in metastatic cases; *n* = 19. Dashed lines represent cases with no significant variation. (**D**) Id. in neoadjuvant setting; *n* = 24. (**E**) Kaplan-Meier analysis of PFS and (**F**) OS in the cohort of 58 HER2-positive metastatic breast cancer patients. YAP1 overexpression (dashed line) was associated with lower PFS (*p* < 0.001) and OS (*p* < 0.001). (**G**) Kaplan-Meier analysis of PFS and (**H**) OS in the same cohort of 58 HER2-positive metastatic breast cancer patients with respect to TAZ overexpression (dashed line). TAZ was associated with lower PFS (*p* = 0.020) and OS (*p* = 0.024).

To understand the clinical implications of these findings, we investigated the prevalence and clinical significance of YAP1 and TAZ overexpression. To do this, we quantified both markers’ expression in a cohort of 58 patients with advanced/metastatic HER2-positive breast cancer previously treated with trastuzumab. When present, both YAP1/TAZ were diffusely distributed throughout the tumor (Figure 7A), with primary expression located in the cytoplasm of tumor cells and nuclear staining in some cases. Nuclear staining was considered as a surrogate of YAP1/TAZ activity status. Weak levels of YAP1/TAZ were detected in normal breast epithelium and stromal cells. We defined the optimal overexpression threshold for YAP1 and TAZ by ROC analysis for every case based on the progression endpoint, and the cutoff was established at 20% of tumor cells (Figure S11). Breast tumors from metastatic patients previously exposed to trastuzumab showed a high incidence of YAP1 (21%) and TAZ (24%) overexpression. The elevated expression of the markers was not dependent on tumor grade, receptor status, or proliferation rate of the tumors. The description of the clinical parameters of these series is included in Table S4. The multivariate survival analysis using Cox’s regression model, which included hormonal status, ER status and YAP1 expression category (low or high), identified YAP1 overexpression as an independent indicator of bad prognosis in the cohort of HER2+ metastatic breast cancer patients (*p* < 0.01) (Table S5).

The observation of YAP1/TAZ relevance after trastuzumab exposure on acquired resistance was also supported by evaluating the modulation of YAP1 and TAZ in the cohorts of patients in whom progression of the disease had been detected, and for whom both a pre-treatment diagnostic and a metastatic after-treatment samples were available (Figure 7B). Of these, 19 cases were selected from the cohort of metastatic patients, showing that 37% of the post-treatment samples presented a significant increase in YAP1/TAZ expression (*p* = 0.003), while the rest of the paired samples did not change their YAP1/TAZ expression levels (no sample showed a decrease in YAP1/TAZ expression after treatment) (Figure 7C). Similarly, 24 pairs were obtained from patients with early HER2-positive breast cancer treated in a neoadjuvant regimen (Figure S12), revealing a significant increase in YAP1/TAZ expression: 75% of post-treatment samples with residual tumor showed increased YAP1/TAZ expression as compared to their pre-treatment samples (*p* = 0.001), whereas the remainder did not vary (Figure 7D).

Finally, based on the upregulation of YAP1/TAZ expression after trastuzumab treatment, we investigated the clinical significance of YAP1/TAZ expression on acquired trastuzumab resistance in metastatic HER2-positive breast cancer patients. Increased YAP1 expression was significantly correlated with worse prognosis, both in terms of PFS (*p* < 0.001, Figure 7E) and OS (*p* < 0.001, Figure 7F). Moreover, breast-cancer cases with upregulation of YAP1 expression after treatment were statistically associated with a shorter life expectancy (OS, *p* = 0.023, Figure S13). The results for TAZ were similar (Figure 7G,H), due to the high correlation of the expression of both markers (Table S4).

## 3. Discussion

Current therapeutic strategies directed against HER2-positive breast cancer are mostly based on newly designed anticancer agents targeting cell-signaling receptors and pathways. Although advances in therapy for HER2-positive breast-cancer patients have led to significant increases in OS and PFS rates, the increasing incidence of acquired resistance to current anti-HER2 directed therapies remains a problem for HER2-positive advanced breast cancer [3]. In particular, trastuzumab resistance seems to be governed by molecular and cellular heterogeneity in HER2-positive breast-cancer cells [20].

Different mechanisms have been proposed to explain the acquisition of resistance to trastuzumab in patients with HER2-positive breast cancer [20]. In a previous report, we generated trastuzumab-resistant breast-cancer cell lines using HER2-positive BT-474 and three other cell lines to identify and target the key nodes of trastuzumab resistance [17]. In our model of BT-474.r2T breast cancer cells, the initial characterization of the molecular alterations found in the HER family proteins as a consequence of the generation of resistance to trastuzumab revealed increased levels of some of the receptors, as well as activation (by phosphorylation) of most of them, including HER2. Although further trastuzumab exposure did result in reduced HER-mediated signaling in all cases, resistance was not abolished. Simultaneously, we found indications of EMT, as well as an increase in the level of CD24, which may be indicative of interconversion after EMT [21], along with and increased migration. These observations agree with the acquisition of resistance, as well as with the further findings of YAP1 activation. Consequently, mechanisms other than HER signaling must be responsible for the increased proliferation and oncogenic potential of the BT-474.r2T cells. 

Our results reveal a novel mechanism of acquired trastuzumab resistance mediated by two pivotal effectors of the Hippo tumor suppressor pathway: the transcriptional co-activator YAP1 and the transcription factors TEAD1-2. When therapeutic pressure is high and continuous, the cell reacts by changing the YAP1 status to its unphosphorylated form, and therefore the protein is translocated to the nucleus, thereby modulating the resistant phenotype. Recent research supports the role of Hippo signaling in the modulation of cancer-cell proliferation and human tumorigenesis by transcriptional regulation of several cell proliferation-related genes, such as *AREG*, *AXL*, *CTGF*, *CYR61*, and *VEGFA* [22,23,24]. According to this, previous research suggested targeting the complex YAP/TEAD as a potential therapeutic target to inhibit the effect of YAP1 deregulation on hepatocarcinoma progression [15]. However, to date the role of Hippo pathway deregulation in development of resistance to anti-HER2 targeted therapies against HER2-positive breast cancer was unknown.

The results obtained using gene expression arrays and phosphoproteome analyses allowed us to identify relevant components of the Hippo signaling pathway as potential biomarkers of acquired resistance to trastuzumab in human HER2-positive breast-cancer cells. YAP1 is a transcriptional cofactor that functions as the main effector of the Hippo signaling pathway, in combination with any member of the TEAD transcription factor family. A known mechanism of negative regulation of the Hippo pathway involves the E3 ubiquitin-protein ligase Itchy homolog (ITCH), which mediates Large Tumor Suppressor 1 (LATS1) ubiquitination and proteasomal degradation, leading to stabilization of dephosphorylated YAP/TAZ, and associating with tumorigenesis in several preclinical models including those of breast tumors [25,26]. In particular, the YAP1 subcellular location and co-activator function are both regulated by phosphorylation [27]. Phosphorylation is the most widely studied protein post-translational modification in biological systems, since it mediates crucial processes such as cell growth, proliferation, and survival. In our model, trastuzumab-resistant BT-474.r2T cells display enriched nuclear location of non-phosphorylated YAP1, since the protein has been dephosphorylated and thus has been translocated from the cytoplasm. At the same time, they exhibit total YAP1 levels lower than sensitive BT-474 cells, since cytoplasmatic retention of YAP1 marks it for degradation [27]. This coincides with higher levels of TEAD2 gene expression and protein abundance in resistant cells. Because of this double YAP1/TEAD2 alteration, we found increased gene expression for their targets *AREG*, *CTGF*, *VEGFA*, and *AXL* (but not *CYR61*). From a physiological point of view, the resistant cells show significantly increased levels of elevated nuclear active form of YAP1 and TEAD compared to parental BT-474 cells and consequently exhibit changes in proliferation, clonogenic capacity, apoptosis, and drug resistance.

A possible mechanism of action suggests that activated HER4 would interact with YAP1 to induce Hippo pathway target genes, according to previous reports [28]. Although HER4 was initially proposed as the dedicated receptor for the mammalian Hippo pathway in the context of neuregulin activation, we argue that this noncanonical signaling would be activated in our BT-474.r2T model as a consequence of a more extensive cross-talk between the EGFR signaling axis and the Hippo-YAP network. Our results showed that HER proteins were overexpressed and phosphorylated following the acquisition of resistance to trastuzumab (Figure 1A). Thus, activation of HER4 (and possibly of HER3, as well) by phosphorylation would trigger YAP1 to induce Hippo target genes, as we confirmed at the transcript and protein levels. Additionally, potential activity of EGFR (also phosphorylated in BT-474.r2T), increased by the positive feedback loop of AREG activation [6], may explain the enhanced YAP1 signaling that we observed in the context of a more aggressive behavior of these resistant cells. This cross-talk between HER family and Hippo-YAP1 networks holds numerous implications for tackling the resistance to trastuzumab in breast-cancer cells.

Specific modulation of the YAP1/TEAD complex should only affect their direct targets, rather than other components upstream of the Hippo pathway, thereby inducing fewer side effects than inhibition of membrane receptors or upstream kinases [18]. Furthermore, exogenous downregulation of YAP/TEAD1-2 activity by RNA-dependent gene silencing restored sensitivity of BT-474.r2T cells to the antiproliferative effect of physiological-equivalent doses of trastuzumab and downregulated transcription of several targeted genes. Conversely, modulation of the Hippo pathway upstream kinase regulators might lead to deregulation of crosstalk with other signaling pathways, thus inducing unexpected side effects. Interestingly, it has been recently reported that TAZ and YAP1 enhance PD-L1 levels in breast-cancer cell lines [29]. The transcriptional activity of the complex TAZ/YAP1/TEAD increases *PD-L1* promoter activity, thereby determining PD-L1 expression and functioning. At the same time, it has become evident in the literature that YAP1 can modify the tumor microenvironment by cytokine upregulation [30]. This matches our recent findings that CCL5 and other cytokines also play a crucial role as mediators of acquired resistance to trastuzumab in our BT-474.r2T model (in press). All these findings suggest the significant impact that the Hippo signaling pathway activity may hold in human cancer immune evasion.

A porphyrin molecule named verteporfin was identified as a strong inhibitor of the YAP1/TEAD complex formation [15]. Recent studies have also demonstrated that verteporfin can inhibit cell growth and induce apoptosis and G0/G1-phase cell cycle arrest in human cancer cells without light activation [31], and although cytotoxic and antiproliferative effects have been reported independently of its effect on YAP [32], verteporfin is considered a promising chemotherapeutic agent for the treatment of cancer. Interestingly, our study demonstrates a combination of verteporfin with trastuzumab can restore trastuzumab sensitivity in BT-474.r2T cells in vitro, decreasing the proliferation rate of trastuzumab-resistant BT-474.r2T cells and downregulating *CTGF* transcription in BT-474.r2T cells. Finally, we proved that disrupting YAP1/TEAD-mediated signaling by treatment with trastuzumab in combination with verteporfin induced growth inhibition and apoptosis of trastuzumab-resistant human xenografts in vivo. These results suggest that verteporfin might play a key suppressing role in controlling trastuzumab-resistant tumors. To summarize, our data suggest that molecular subclassification of HER2-positive breast-cancer patients and the development of a combination therapy targeting the Hippo pathway effectors might significantly counteract trastuzumab resistance and improve therapeutic outcomes in HER2-positive breast-cancer patients.

## 4. Material and Methods

### 4.1. Cell Cultures and Treatments

BT-474 (ATCC HTB-20^TM^) cell line was authenticated (LGC Standards; tracking no: 710259498) and cultured at 37 °C in 5% CO_2,_ in DMEM/F12 medium (Sigma Aldrich Spain, Madrid, Spain), supplemented with 10% fetal bovine serum (FBS) (Life Technologies S.A., Alcobendas, Spain), L-glutamine (200 mM) (Gibco, Thermo Fisher Scientific ES, Madrid, Spain), penicillin G (100 U/mL), and streptomycin (100 μg/mL) (Gibco). A trastuzumab-resistant BT-474 cell line was generated by continuous exposure to trastuzumab as previously described [17]. Briefly, BT-474 cells were grown in 10 μg/mL trastuzumab for 4 weeks, and 15 μg/mL of trastuzumab for 8 months until the resulting cells (BT-474.r2T clone) were defined as resistant to trastuzumab treatment. Once the establishment of resistance had been confirmed, the cells were cultured with a 15-μg/mL maintenance dose. The resistance rate was determined monthly by cell proliferation assays, and using the algorithm described by O’Brien et al. that estimates the correlation between the growth rates of treated and untreated cells, based on cell-doubling time. Cells with ≥ 1.2-fold growth were considered responsive to the treatment [33]. All cells used for the experiments were free of mycoplasma contamination as assessed by previously a described protocol [17]. Reagents: recombinant humanized monoclonal HER2 antibody trastuzumab (a concentration of 15 µg/mL was selected as indicated elsewhere [17]) (Herceptin, Genentech, San Francisco, CA, United States); verteporfin (Sellekchem Spain, Madrid, Spain).

### 4.2. Transwell Migration Assay

Migration assays were performed using 24-well plates with transwell permeable supports of 6.5 mm insert and a polycarbonate membrane with an 8 µm pore size (Costar #3422, Corning, Madrid, Spain). Cells were seeded in the upper chamber at 2 × 10^4^ cells/mL in 0.1 mL of serum-free RPMI-1640 media. A volume of 0.8 mL of media supplemented with 10% FBS was placed in the bottom well as a chemo-attractant. After incubation for 24 h at 37 °C in an atmosphere containing 5% CO2, migrated cells on the lower surface were stained using crystal violet and counted under a light microscope. 

### 4.3. Microarray Processing 

Total RNA from BT-474 and BT-474.r2T cells (either untreated or treated with 15 µg/µL trastuzumab for 48 h) was used for gene expression profiling. RNA extracts were isolated using the RNeasy Mini kit (Qiagen Iberia S.L., Madrid, Spain), following the manufacturer’s instructions. RNA purity and integrity were assessed using NanoDrop 2000 (Thermo Fisher Scientific), as well as the Agilent 2100 Bioanalyzer (Agilent Technologies Spain, Madrid, Spain). All RNA samples yielded a high purity (A260/280 > 2.0 and A260/230 > 1.4), and a high RNA integrity number (RIN > 9.4) and were consequently used for microarray analysis. Microarray expression profiles were obtained with the Affymetrix Genechip^®^ Human Gene 2.0 ST (Affymetrix, Thermo Fisher Scientific). Preparation of cDNA, hybridization, and microarray processing were performed according to standard manufacturer protocols (Affymetrix). Following hybridization, the arrays were stained in the Affymetrix GeneChip Fluidics Station 450 and scanned using a GeneChip Scanner 3000 7G. Differentially expressed genes were determined by microarray analyses using biological duplicates from independent experiments. Data were processed following the methodology previously described [34]. All microarray procedures were performed at the IMIM Microarray Analysis Service Core Facility (Barcelona, Spain).

### 4.4. Microarray Data Analysis

Primary data analysis was performed with the robust multiarray average (RMA) method for normalization, implemented in the oligo v.1.38 package [35], and hugene20sttranscriptcluster.db v.8.5.0 for gene annotation from Bioconductor (https://www.bioconductor.org) [36]. The expression value of genes with several probes was taken as the mean of the expression values of all those probes. The principal-component analysis (PCA) was plotted using the pca2d method from the R package pca3d. Differential gene expression between groups of cells (baseline BT-474.r2T vs. BT-474, and trastuzumab-treated BT-474.r2T vs. BT-474) was assessed using the limma package from Bioconductor [37], using the Benjamin-Hochberg false discovery rate (FDR) method for multiple testing correction as implemented in the Babelomics portal. Genes with FDR-adjusted *p* < 0.05 were taken as significant. Fold change (FC), defined as a difference in log2 mean values between experimental groups, was also calculated using Babelomics, selecting genes with a FC >2-fold. Data are available through the Gene Expression Omnibus under dataset identifier GSE89216.

### 4.5. Gene Set Enrichment Analysis

Gene set enrichment analysis (GSEA) [38] was performed. Functional enrichment was applied using annotations from the MsigDB, Reactome, KEGG, and NCI databases. Genes were ranked based on the limma moderated t-statistic. After Kolmogorov-Smirnoff testing, those gene sets showing FDR < 0.05 were considered enriched between classes under comparison.

### 4.6. Stable Isotope Labeling Using Amino Acids in Cell Culture (SILAC) Labeling and Enrichment in Phosphopeptides

When implementing the SILAC labeling strategy, culture media were supplemented with unlabeled 0.398 mM Arg0 and 0.798 mM Lys0 and with labeled 0.398 mM Arg6 and 0.798 mM Lys6 (Silantes GmbH, Munich, Germany), for light medium and heavy medium, respectively. Figure S14 shows the experimental workflow for the SILAC experiment. After five passages, labeled BT-474 cells were treated with 15 µg/mL trastuzumab, while unlabeled BT-474 cells remained untreated; conversely, unlabeled BT-474.r2T cells were supplemented with 15 µg/mL trastuzumab, while labeled BT-474.r2T cells remained untreated. After 0 minutes, 12 minutes, and 7 days of trastuzumab exposure, protein extraction and quantification were performed. The following protein enrichment step was performed in the Proteomics Unit of Complutense University of Madrid (Madrid, Spain), which belongs to the ProteoRed network (Spain). Protein digestion was performed as reported elsewhere, with some modifications [39]. Briefly, the light- and heavy-cell lysates were combined at a 1:1 ratio (w/w). Six hundred µg of the mixed heavy/light protein samples were denatured, filtered, alkylated, and finally digested with sequence grade-modified trypsin (Roche España, Madrid, Spain) at an enzyme-to-substrate ratio of 1:50. Phosphopeptide enrichment by sequential elution from IMAC (SIMAC) [40] was done as previously described [39]. The IMAC flow-through and the IMAC eluted acidic fraction were further enriched for phosphopeptides through a TiO2 microcolumn.

### 4.7. Mass Spectrometry Analysis (RP-LC-MS/MS) and Protein Identification and Quantification

The analysis was carried out in the CBMSO Proteomics Core Facility (Madrid, Spain), which belongs to ProteoRed. The desalted protein digest was dried, resuspended in 10 μL of 0.1% formic acid, and analyzed by RP-LC-MS/MS in an Easy-nLC II system coupled to an LTQ-Orbitrap-Velos-Pro hybrid mass spectrometer (Thermo Fisher Scientific). Briefly, peptides were concentrated (on-line) by reverse phase chromatography using a 0.1mm × 20 mm C18 RP precolumn (Thermo Fisher Scientific), and then separated using a 0.075 mm × 250 mm C18 RP column (Thermo Fisher Scientific) operating at 0.3 μL/min. Peptides were eluted using a 240 min dual gradient: from 5% to 25% solvent B in 180 min followed by 25% to 40% solvent B to in 60 min (Solvent A: 0,1% formic acid in water, solvent B: 0,1% formic acid, 80% acetonitrile in water). Peptides were ionized using a 30 μm ID stainless steel nano-bore emitter (Proxeon, Odense, Denmark). Full-scan spectra were acquired in the Orbitrap analyzer at a resolution of 30.000. Peptides were detected in survey scans from 400 to 1600 amu (1 micro-scan), followed by fifteen data dependent MS/MS scans (Top 15), using an isolation width of 2 m/z, a normalized collision energy of 35%, and dynamic exclusion for 30 seconds. MS/MS spectra were searched against the Uniprot human reference proteome FASTA file (release Mar 2017; 42,161 entries) using MaxQuant 1.5.4.1 software [41]. The following settings were used: a multiplicity of 2, and a maximum of 3 labeled aminoacids per peptide and Lys-6 and Arg-6 were selected as heavy labels. Cysteine carboxamidomethylation was treated as fixed modification, and methionine oxidation and N-terminal acetylation as variable modifications. Peptide precursor ions were searched with a maximum mass deviation of 6 ppm and fragment ions with a maximum mass deviation of 20 ppm. Peptide, protein, and site identifications were filtered at an FDR of 1% using the decoy database strategy. Changes in phosphorylation were determined by analysis of phosphosite intensity values and heavy/light (H/L) ratio of phosphosites in three experiments were determined. Only class-1 phosphosites (with a localization probability score >0.75) were considered. The normalized H/L ratio was inverted (L/H) for conditions in which the light state (Lys-0/Arg-0) is the respective sample, while the heavy state (Lys-6/Arg-6) is the reference. 10^(LOG10) or –1/10^(LOG10) transformation was performed to get positive/negative values, where 1 = no change, and SILAC ratio = 1.5 was determined as cutoff. Multiple phosphosites were reported in cases where multiple phosphorylations were identified per phosphoprotein. The minimal peptide length was 7 amino acids and the minimum Andromeda [42] score for modified peptides was 40 and the corresponding minimum delta score was 6. Proteins that could not be differentiated based on MS/MS spectra alone were separated into protein groups (default MaxQuant settings). The mass spectrometry proteomics data have been deposited to the ProteomeXchange Consortium via the PRIDE [43] partner repository with the dataset identifier PXD010574.

### 4.8. Signaling Network and Gene Ontology Analysis

Gene identifiers mapped for proteins of interest (HGNC) were uploaded to the STRING v10.5 webtool [44] for retrieval of protein-protein interaction (PPI) information using default settings. This information was then imported into Cytoscape 3.4.0 [45] for visualization and further analysis with the Phosphopath Cytoscape app [46]. Enriched gene ontology (GO) terms (relative to the whole human proteome) for selected protein subsets were retrieved by applying the STRING plugin, using default hypergeometric statistics with Benjamini-Hochberg FDR correction for multiple testing. Full lists of significant GO terms, including differentially regulated candidates that map to each term, are presented in Table S3.

### 4.9. Quantitative Real-Time RT-PCR

Total RNA extracts were isolated using RNeasy Mini kit. RNA purity and integrity were assessed by spectrophotometric determination (NanoDrop ND-2000). RNA was reverse-transcribed to complementary DNA (cDNA) using the Universal Transcriptor cDNA synthesis kit (Roche). Quantitative PCR (qPCR) cDNA amplification was performed in a LightCycler 480 system using Universal Probe Library assays (Roche) specific for *AREG*, *CTGF*, *CYR61*, *TAZ*, *TEAD1*-4, *VEGFA*, and *YAP1* (Table S6). Relative gene expression was calculated according to the comparative cycle threshold (Ct) method [47]. Expression of *ATP5E* was used for normalization, and results were plotted as relative mRNA expression levels normalized to the internal control.

### 4.10. Western Blotting (WB) Analysis

Total protein extracts were isolated with RIPA buffer containing 1× cOmplete™ protease inhibitor cocktail tablets and 1× PhosStop™ phosphatase inhibitor cocktail tablets (Roche) at 4 °C for 20 min. Nuclear and cytosolic protein fraction lysates were isolated using the K266-25 Nuclear/Cytosol Fractionation Kit (BioVision, Milpitas, CA, USA), according to manufacturer indications. Protein extracts were clarified (13,000 × g, 10 min, 4 °C), denatured, and subjected to SDS-PAGE and WB. Primary antibodies: TAZ (#4883), TEAD1 (#12292), YAP1 (#14074), pYAP1-Ser109 (#46931) pEGFR-Tyr1173 (#4407), EGFR (#4267), pHER2-TyrY1221/1222 (#2243), HER2 (#2242), pHER3-Tyr1289) (#4791), pHER4-Tyr1284 (#4757), HER4 (#4795), N-cadherin (#D4R1H), E-cadherin (#3195S) (1:1000), (Cell Signaling Technology, USA); lamin B1 (#ab16048), fibronectin (#ab2413) (1:1000), (Abcam); β-actin (1:10000) (#A5441), CTGF (#HPA031075) (1:250), GAPDH (#G8795) (1:10000), and TEAD2 (#SAB4503373), CD24 (#SAB4700624), occludin (#SAB4200593) (Sigma Aldrich); HER3 (#MA5-13053) (1:1000) (Invitrogen); vimentin (#550513BD) (1:1000) (Pharmigen). Proteins were detected with secondary antibodies conjugated to alkaline phosphatase (1:5000) (Sigma Aldrich) by chemoluminescence with TROPIX CSPD Substrate and Nitro-Block-II Enhancer (Applied Biosystems, Thermo Fisher Scientific). Original WB images and densitometry analyses are provided as Supplementary Material (Figure S15).

### 4.11. siRNA Silencing

BT-474.r2T cells were transfected with 50 nM/well smart-pool siRNAs targeting YAP1, TEAD1, or TEAD2 (Dharmacon Products) dissolved in a mixture of Opti-MEM Medium and Lipofectamine 2000 (Invitrogen, Thermo Fisher Scientific) for 16 h. For long-term experiments, and to avoid the loss of effect due to the transitory nature of silencing, a second transfection was performed 72 h after the initial one. In parallel, a transfection with a scrambled (SCR) siRNA was performed under the same conditions. After transfection, cell-culture medium was replaced, BT-474.r2T cells were treated with 15 µg/mL trastuzumab for 7 days, and finally cell growth was assessed as described above. For gene and protein expression analysis, cells were subjected to siRNA, and after 48 h they were lysed and subjected to qPCR and WB assays as described above.

### 4.12. Establishment of Resistant Xenografts in a Murine Model and Preclinical Study

All animal studies were performed at the PRBB Animal Facility (Barcelona, Spain), in accordance with institutional ethical guidelines. Five-week-old female severe combined immunodeficiency (SCID/beige) mice (Charles River Laboratories España, Barcelona, Spain) were subcutaneously inoculated in their flank with 2.5 × 10^6^ BT-474.r2T cells mixed with Matrigel as previously described [17]. Once the average volume of tumors reached 100 mm^3^, mice were randomly allocated into four groups of five mice each. For therapeutic studies, the concentration and time of treatments was inspired from previous reports in the literature [48,49] and administered as follows: (i) in the first group, mice received control treatment with human IgG1 ĸ (10 mg/kg, Sigma Aldrich); (ii) the second group received trastuzumab (10 mg/kg); (iii) in the third group, mice were treated with verteporfin (40 mg/kg); and (iv) the fourth group received the combination of trastuzumab and verteporfin (10 mg/kg and 40 mg/kg, respectively). All treatments were prepared in 8% DMSO and injected intraperitoneally (i.p.) every other day for three weeks. Tumor diameters were serially measured with digital calipers, and tumor volumes were calculated using the following formula: volume = width^2^ × length/2. After three weeks, tumor xenografts obtained from BT-474.r2T cells were excised and included in formalin-fixed paraffin-embedded (FFPE) blocks.

### 4.13. Immunohistochemistry (IHC)

FFPE 3-µm sections were obtained from paraffin-embedded human tumor xenografts in mice and human tumors, and IHC was performed as previously described [50]. Briefly, FFPE sections were placed on positively charged glass slides on a Dako Link platform (Dako, Agilent Technologies Spain, Madrid, Spain). After deparaffinization, heat antigen retrieval was performed in a pH 9 EDTA-based buffered solution (Dako). Endogenous peroxidase was quenched. Primary antibodies (all from Cell Signaling) against YAP1 (#14074), TAZ (#1674883/v386), cleaved caspase3 (c-casp3) (1:100), and phospho-Histone3-Ser10 (pH3) (1:100) were used, and HER2 status was assessed by HercepTest (K5207; Dako). Antigen–antibody reaction was detected by incubation with an anti-mouse/rabbit Ig-dextran polymer coupled with peroxidase (Flex+, Dako). Sections were then visualized with 3,3′-diaminobenzidine and counterstained with hematoxylin. All immunohistochemical staining was performed on a Dako Autostainer platform. A semiquantitative estimation of the percentage of tumor cells positively stained for YAP1 and TAZ nuclear expression was calculated by a pathologist (FR), and the results ranged from 0 to 100. Proper positive and negative controls were assayed to validate the staining procedure (Figure S16).

### 4.14. Analysis of TCGA Samples

The publicly available clinical data and annotations for a series of 1097 breast cancer patients were directly downloaded from the TCGA and the Genomic Data Commons (GDC) data portal [51] (http://cancergenome.nih.gov, January 2015), using the R package in TCGA-Assembler [52]. Clinical data for breast cancer samples were obtained from the original clinical dataset (nationwidechildrens.org_clinical_patient_brca.txt) as described in previous studies [51]. We classified the TCGA breast cancer tumors into two groups based on their HER2 status. 71 cases (6.5%) that overexpressed HER2 and received adjuvant chemotherapy with trastuzumab were considered the group of interest; 1026 cases (93.5%) were not included in this study, because they did not overexpress HER2. 

### 4.15. Patients and Tumor Samples

A single-institution retrospective analysis including 58 HER2-positive metastatic breast cancer patients from Fundación Jiménez Díaz Biobank (Madrid) diagnosed between 2000 and 2014 was carried out, including clinical follow-up (median: 3.9 years), in accordance with current legislation. In all cases, representative FFPE samples of the infiltrating primary tumor component were available. Also, paired pre- and post-trastuzumab samples were available for 19 cases of relapse or disease progression. Clinical data and clinical-pathological information were collected from all cases.

### 4.16. Statistical Analysis

Statistical analysis was carried out with the IBM SPSS Statistics package, version 21.0. Overexpression criteria were defined by receiver operating characteristic (ROC) curve for YAP1 and TAZ proteins. Overall survival (OS) was defined as the time elapsed from the date of initial diagnosis of metastatic disease to the date of death from any cause or the date of last follow-up. Progression-free survival (PFS) was defined as the time from treatment to either progressive disease or death from any cause, recorded at last contact. Survivals were analyzed by the Kaplan-Meier method and curves were compared using the log-rank test. Multivariate analysis, including continuous quantitative and qualitative clinical-pathologic parameters, was carried out using the Cox proportional hazards model. All measured data are expressed as means ± standard deviations of at least three replicates (unless otherwise indicated). Statistical significance was analyzed by a two-tailed Student’s t-test (*: *p* < 0.05, **: *p* < 0.01, ***: *p* < 0.001). This work was performed in accordance with the Reporting Recommendations for Tumor Marker Prognostic Studies (REMARK) guidelines [53].

### 4.17. Availability of Data and Materials

The datasets generated and analyzed during the current study are available in the GEO repository, series GSE89216, and in the ProteomeXchange Consortium via the PRIDE [43] partner repository with the dataset identifier PXD010574.

### 4.18. Ethics Approval and Consent to Participate

This study was conducted in full accordance with the guidelines for Good Clinical Practice and the Declaration of Helsinki. Written informed consent was obtained from each participant. Approval of the protocol and of any amendments was obtained from the ethics committee of the Fundación Jiménez Díaz hospital (Madrid, Spain) (reference number: PIC 13-2016).

All animal studies were performed at the PRBB Animal Facility (Barcelona, Spain), in accordance with institutional ethical guidelines. Approval of the protocol and of any amendments was obtained from the ethics committee of the Ethical Committee for Animal Research of the Barcelona Biomedical Research Park (EEA-PRBB, Barcelona, Spain) (reference number: 5778).

## 5. Conclusions

Using an unbiased global transcriptome and proteomic analysis of HER2-positive breast-cancer cells, we propose a new model in which acquired resistance to trastuzumab may be mediated by the increased activity of the YAP1/TEAD1-2 complex. Taken together, our data support a key role for the YAP co-activator and its molecular interaction partners, i.e., TEAD transcription factors, in acquired resistance to trastuzumab in breast cancer, suggesting that these molecules could be potent druggable targets to prevent treatment resistance to anti-HER2 targeted therapy. YAP1/TEAD inhibitors may therefore have potential as a novel, non-chemotherapeutic treatment strategy when administered in combination with trastuzumab for HER2-positive breast cancer.

## Supplementary Materials

The following are available online at https://www.mdpi.com/2072-6694/12/5/1108/s1, Figure S1: Venn diagram of the top predicted candidate Class 1 phosphosite biomarkers of resistance to trastuzumab, Figure S2: Principal Component Analysis (PCA) of probe-level gene expression data. BT-474 and BT-474.r2T cells were grown at both basal and trastuzumab treatment conditions, and duplicate samples were generated for every condition, Figure S3: Heatmap showing relative gene expression in BT-474.r2T and BT-474 cells, both under baseline conditions, and after treatment with 15 mg/mL trastuzumab for 48 h, Figure S4: Prediction map of molecular interactions involved in our model of acquired resistance to trastuzumab, according to phosphorylation signals of the identified candidates by SILAC phosphoproteomic analysis (overlap within 3 temporary datasets, ≥ 1.5-fold change) and mRNA expression microarrays (BT-474 vs. BT-474.r2T cells, both untreated and treated with trastuzumab, ≥ 2-fold change), Figure S5: Immunoblotting analysis confirmed the reduction in protein abundance levels for YAP1, TEAD1, and TEAD2 after RNA silencing, Figure S6: A. Verteporfin dose-response curve after 7 days of culture in BT-474 (IC50 = 77 nM; IC20 = 15 nM) and BT-474.r2T (IC50 =104 nM; IC20 = 53 nM) cell lines. B. Representative phase contrast images of trastuzumab-resistant BT-474.r2T cells treated at growing concentrations of verteporfin, Figure S7: Combination treatment of trastuzumab plus verteporfin: synergy studies in BT-474 and BT-474.r2T to establish a dual working concentration, Figure S8: Effect of 20 nM verteporfin treatment (in combination with 15 µg/mL trastuzumab) in BT-474 cell line proliferation, after silencing of the different Hippo pathway transducers, Figure S9: Combined treatment of 10 mg/kg trastuzumab with 40 mg/kg verteporfin significantly reduced proliferation and increased apoptosis of trastuzumab-resistant xenograft tumors, Figure S10: OS for a series of TCGA samples from breast primary cancer patients, according to their expression levels of different Hippo pathway effectors, Figure S11: ROC curve in metastatic setting (clinical endpoint: early progression). Cutoff: 20–50% of tumor cells, Figure S12: Representative IHC images of YAP1 staining from A. A paired sample pre-therapy (1) and post-therapy (2) with trastuzumab from a tumor of a patient who progressed to the treatment. B. Neoadjuvant samples from four different breast cancer patients; Figure S13: OS for pre- and post-treatment paired samples from metastatic breast cancer patients, stratified by upregulation or not of YAP1 expression levels, Figure S14: A. Experimental workflow for the SILAC experiment, Figure S15: Original WB images and densitometric analyses, Figure S16: Positive and negative controls for YAP1 IHC. Formalin-fixed and paraffin-embedded pellets were obtained from parental, trastuzumab-sensitive BT-474 cell line (A) and resistant (B) BT-474.r2T cells. IHC assay for YAP1 expression was performed as described, Table S1: Summary table of the proteins identified in the SILAC-based approach to identify differential regulation of the trastuzumab-resistant BT-474.r2T cell line, Table S2: GSEA of the differentially regulated genes in BT-474.r2T cells as compared to BT-474 (Molecular Signatures Database, Oncogenic Signature), Table S3: Overlapping of two out of three time data sets (BH corrected *p*-value ≤ 0.05), Table S4: Correlation between YAP1 and TAZ abundance levels, as determined by IHC, in the cohort of 58 samples, Table S5: Multivariate analysis of YAP1 expression and clinical and pathological indicators, Table S6: List of primers. The forward (FW) and reverse (RV) sequences of each primer used in this study is provided.
