# Supplementary material for: The Hippo Pathway Transducers YAP1/TEAD Induce Acquired Resistance to Trastuzumab in HER2-Positive Breast Cancer
Source: Cancers (Basel). 2020 Apr 29;12(5):1108. doi: 10.3390/cancers12051108 (PMC7281325; doi:10.3390/cancers12051108)
Supplement: Supplementary file 1 [file cancers-12-01108-s001.pdf]

Supplementary Materials: The Hippo Pathway Transducers YAP1/TEAD Induce Acquired Resistance to Trastuzumab in HER2-Positive Breast Cancer

Paula González-Alonso, Sandra Zazo, Ester Martín-Aparicio, Melani Luque, Cristina Chamizo, Marta Sanz-Álvarez, Pablo Minguez, Gonzalo Gómez-López, Ion Cristóbal, Cristina Caramés, Jesús García-Foncillas, Pilar Eroles, Ana Lluch, Oriol Arpí, Ana Rovira, Joan Albanell, Sander R. Piersma, Connie R. Jimenez, Juan Madoz-Gúrpide and Federico Rojo

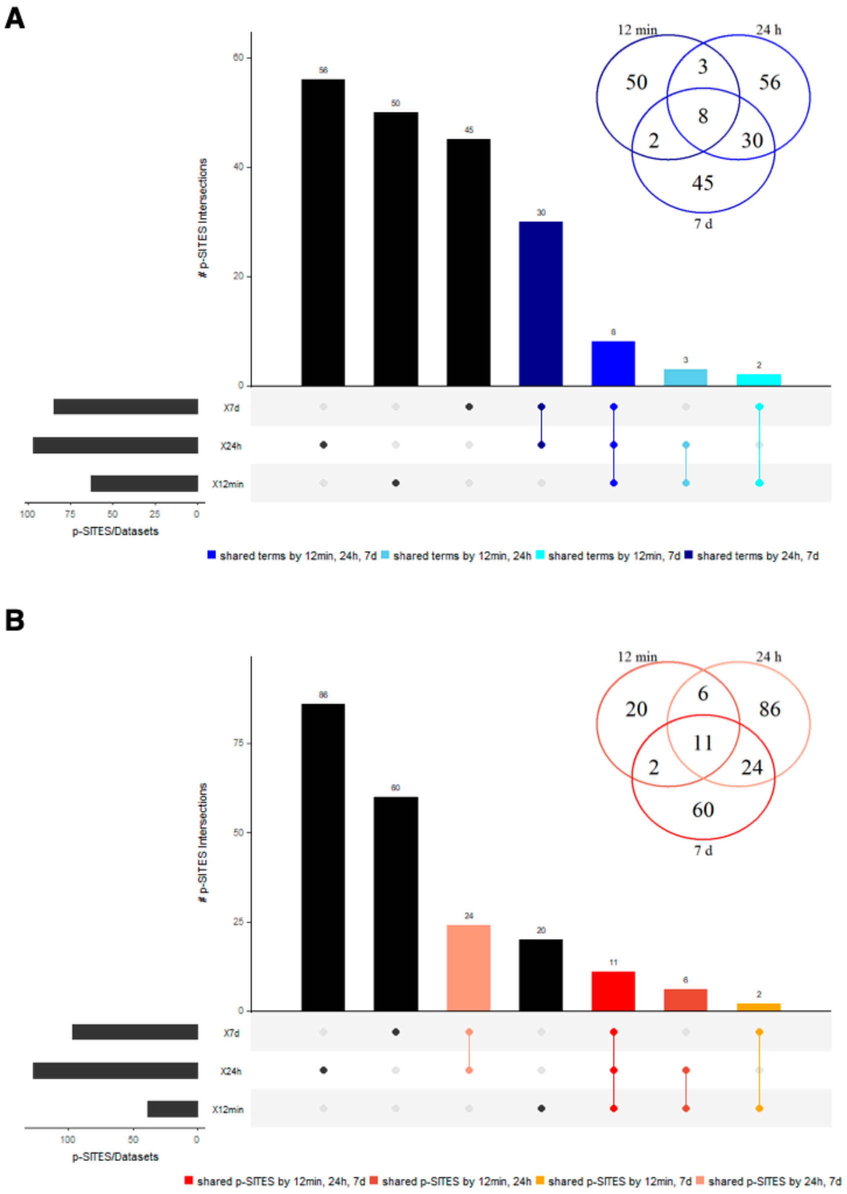

Figure S1. Venn diagram of the top predicted candidate Class 1 phosphosite biomarkers of resistance to trastuzumab. Venn diagrams showing all the partial overlaps between the three time data sets: 12 min, 24 h and 7 days. Four conditions were considered within each data set (overlap among significant regulation in resistant vs. sensitive groups, and treated vs. untreated groups). As predicted by this experimental approach,

overlapping biomarkers in three out of three time data sets are centered in the diagram. Bar graphs show number of candidates from intersections where two sets out of three overlap and those that are unique to each data set. All candidates showed at least ≥1.5-fold differences. **A.** Negative regulation (blue scale). **B.** Positive regulation (red scale).

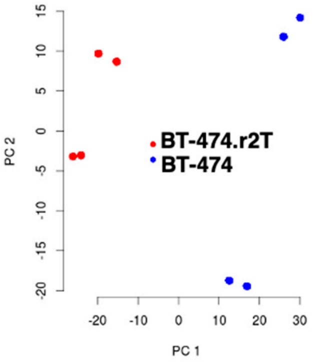

Figure S2. Principal Component Analysis (PCA) of probe-level gene expression data. BT-474 and BT-474.r2T cells were grown at both basal and trastuzumab treatment conditions, and duplicate samples were generated for every condition. PCA showed that the results could be explained from only two main components, one related with the resistance, and the second one with the treatment.

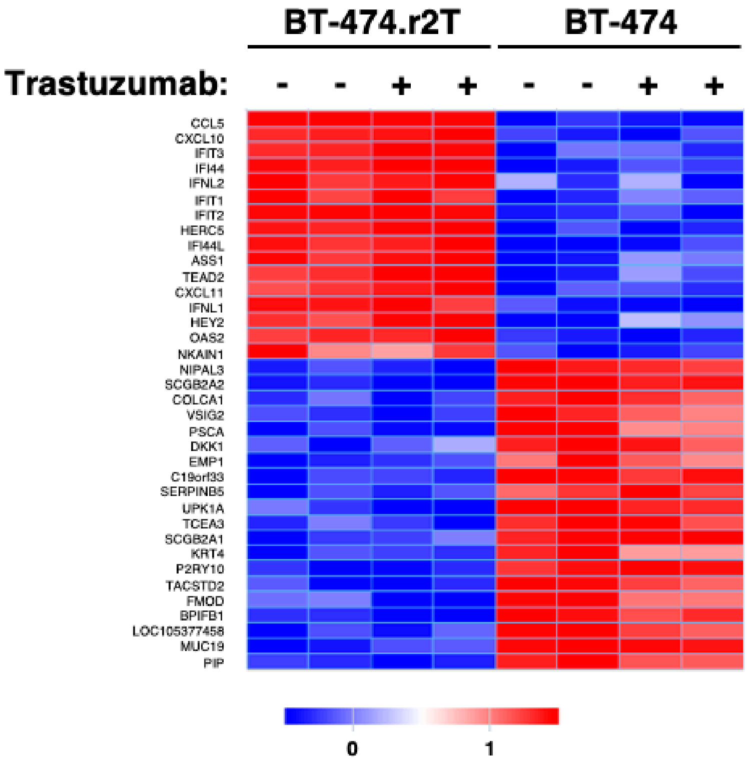

Figure S3. Heatmap showing relative gene expression in BT-474.r2T and BT-474 cells, both under baseline conditions, and after treatment with 15 mg/ml trastuzumab for 48 h. Thirty-six genes showed trastuzumab-independent differential regulation of gene expression in BT-474 and BT-474.r2T cells (16 genes were significantly upregulated in BT-474.r2T cells, whereas 20 genes were significantly downregulated).

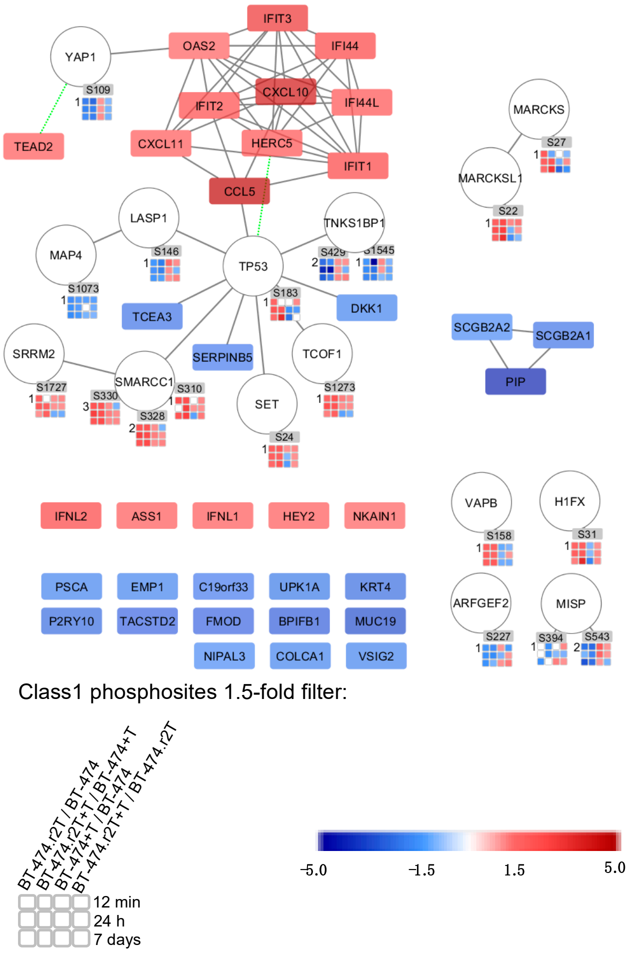

Figure S4. Prediction map of molecular interactions involved in our model of acquired resistance to trastuzumab, according to phosphorylation signals of the identified candidates by SILAC phosphoproteomic analysis (overlap within 3 temporary datasets, ≥ 1.5-fold change) and mRNA expression microarrays (BT-474 vs. BT-474.r2T cells, both untreated and treated with trastuzumab, ≥ 2-fold change). Rectangles represent identified mRNA, and color scale describes change in expression. Ellipses depict proteins; phosphorylated residues are indicated, and small squares show quantification time points for SILAC experiments. Blue scale color shows negative regulation (8 phosphosites, 19 mRNAs); red color describes positive regulation (11 phosphosites, 16 mRNAs); yellow depicts known kinases; white-labelled time points stand for missing values. Class-1 phosphosites were selected for a FC > 1.5.

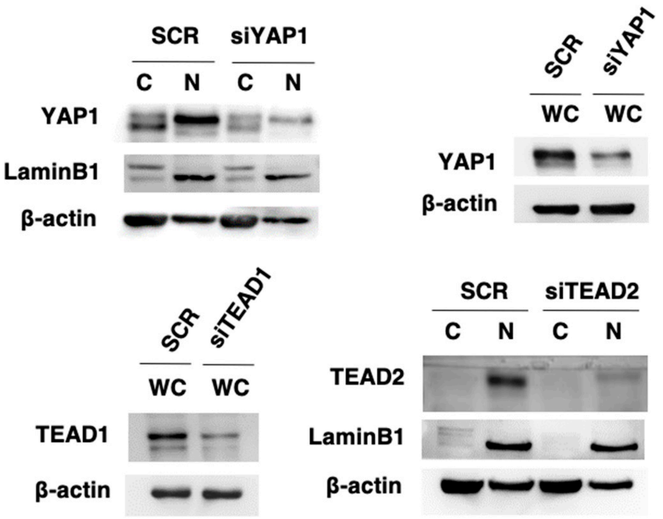

Figure S5. Immunoblotting analysis confirmed the reduction in protein abundance levels for YAP1, TEAD1, and TEAD2 after RNA silencing. The panels show the effects of siRNAs in cytoplasm (C) and nuclear (N) extracts of BT-474.r2T cells, and whole-cell (WC) lysates.

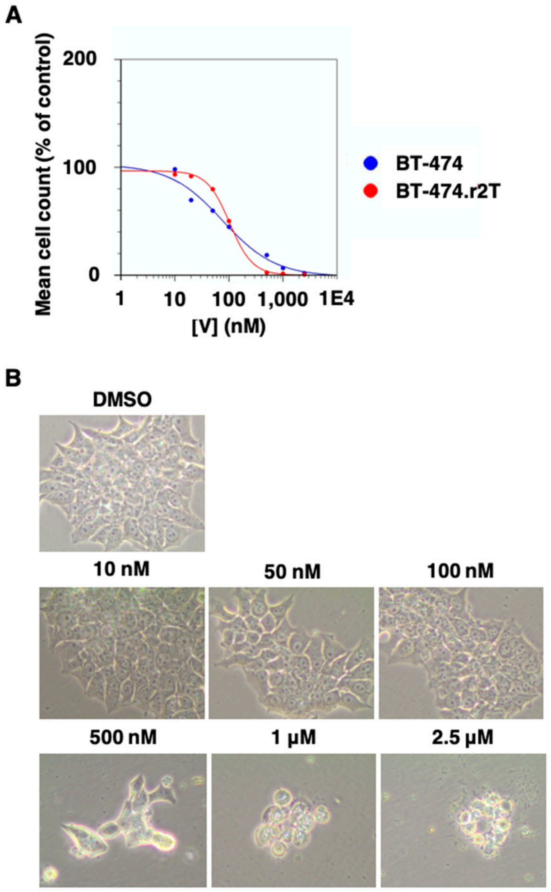

Figure S6. A. Verteporfin dose-response curve after 7 days of culture in BT-474 (IC50 = 77 nM; IC20 = 15 nM) and BT-474.r2T (IC50 =104 nM; IC20 = 53 nM) cell lines. **B.** Representative phase contrast images of trastuzumab-resistant BT-474.r2T cells treated at growing concentrations of verteporfin.

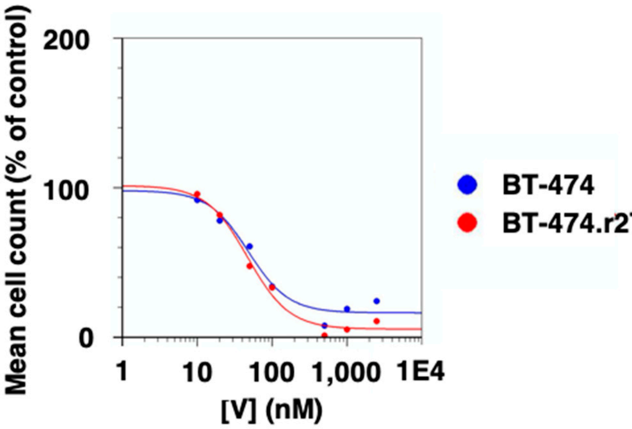

Figure S7. Combination treatment of trastuzumab plus verteporfin: synergy studies in BT-474 and BT-474.r2T to establish a dual working concentration. The verteporfin IC50 was determined in the BT-474 cell line (IC50 = 48 nM), and in resistant BT-474.r2T cell line (IC50 = 33 nM), concomitantly treated with 15 µg/ml trastuzumab.

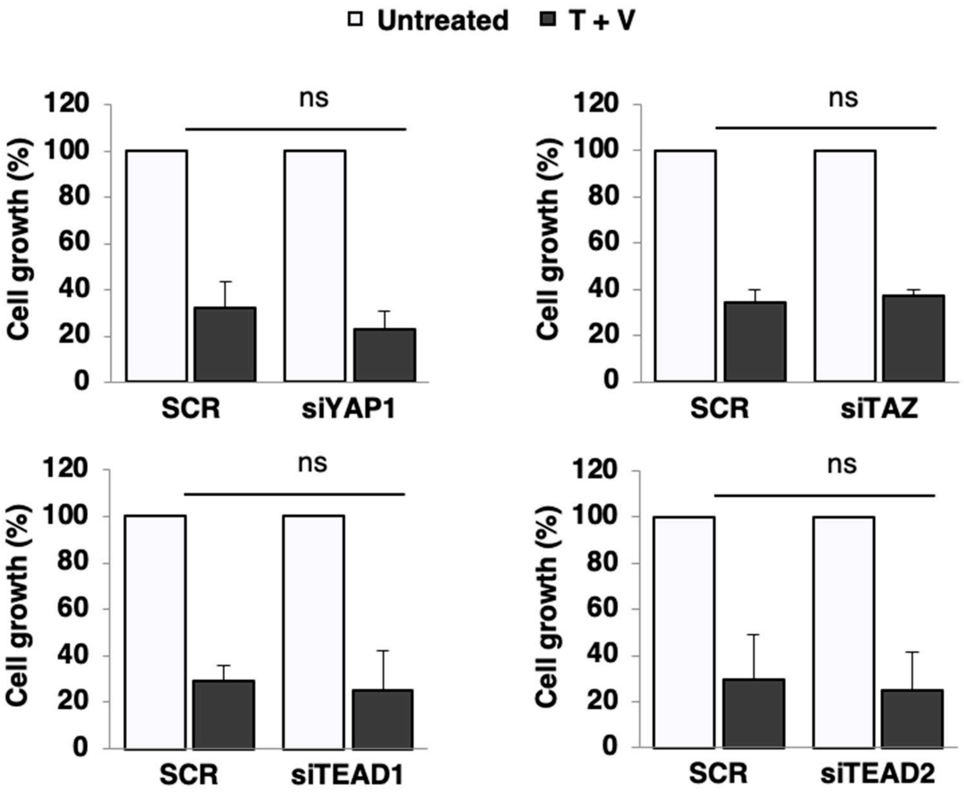

Figure S8. Effect of 20 nM verteporfin treatment (in combination with 15 µg/mL trastuzumab) in BT-474 cell line proliferation, after silencing of the different Hippo pathway transducers.

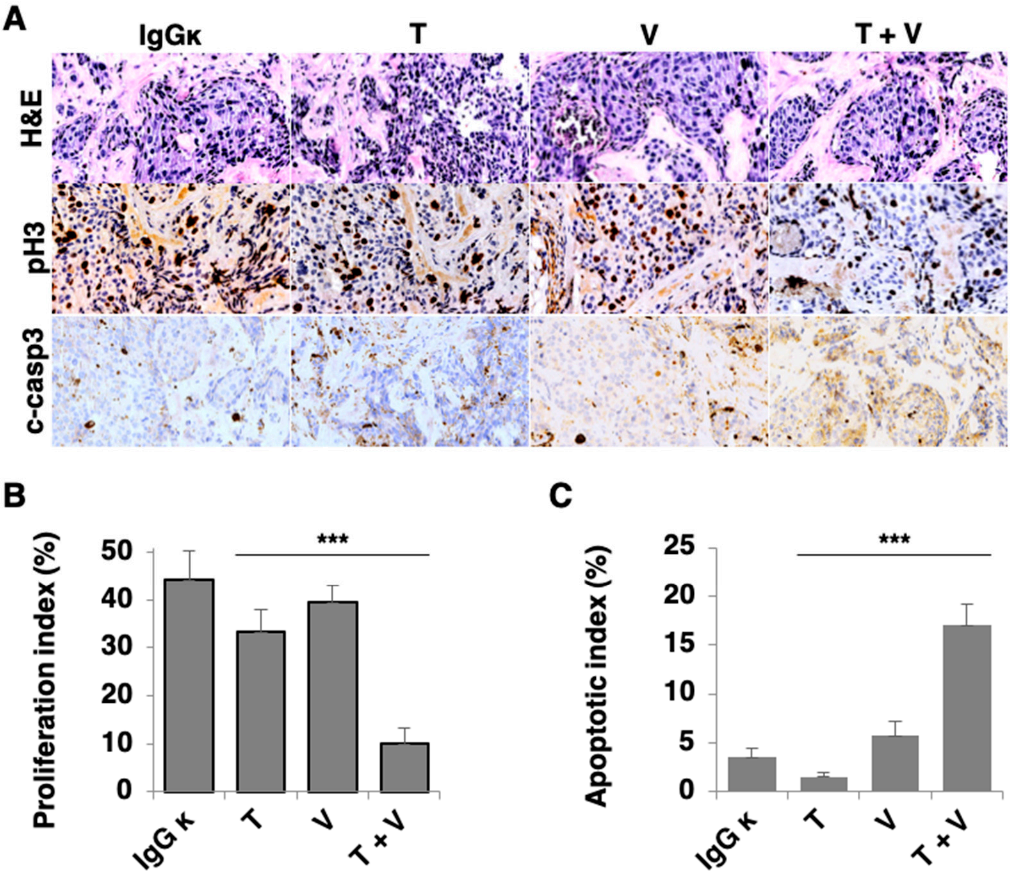

Figure S9. Combined treatment of 10 mg/kg trastuzumab with 40 mg/kg verteporfin significantly reduced proliferation and increased apoptosis of trastuzumab-resistant xenograft tumors. **A.** Representative IHC images of pH3 staining and c-casp3 of FFPE sections from BT-474.r2T xenografts. DAB stain; hematoxylin counterstain (×400 magnification). **B.** IHC detection of pH3 and **C.** c-casp3 expression in tumor samples from the different groups of treatment in the murine model. All results are mean ± s.e.m. values (n = 5).

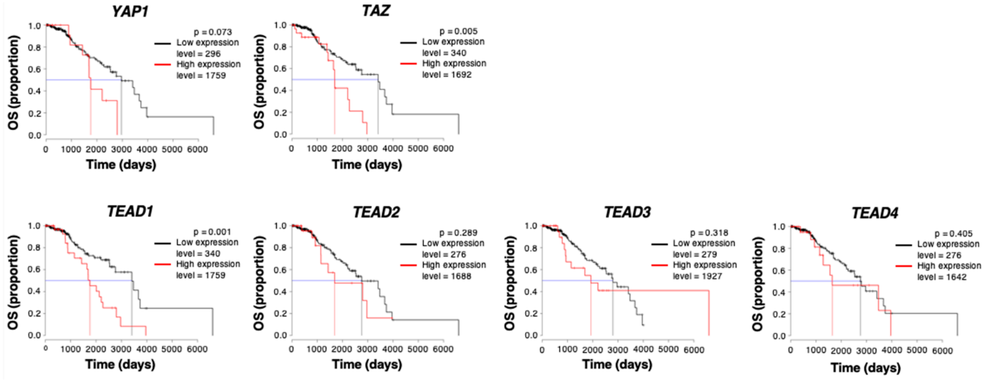

Figure S10. OS for a series of TCGA samples from breast primary cancer patients, according to their expression levels of different Hippo pathway effectors. Median survival is specified for both low and high expression levels for every marker.

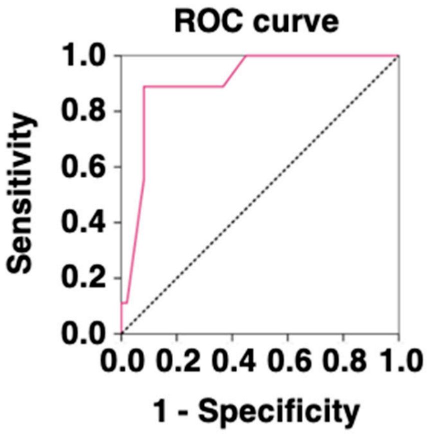

Figure S11. ROC curve in metastatic setting (clinical endpoint: early progression). Cutoff: 20–50% of tumor cells.

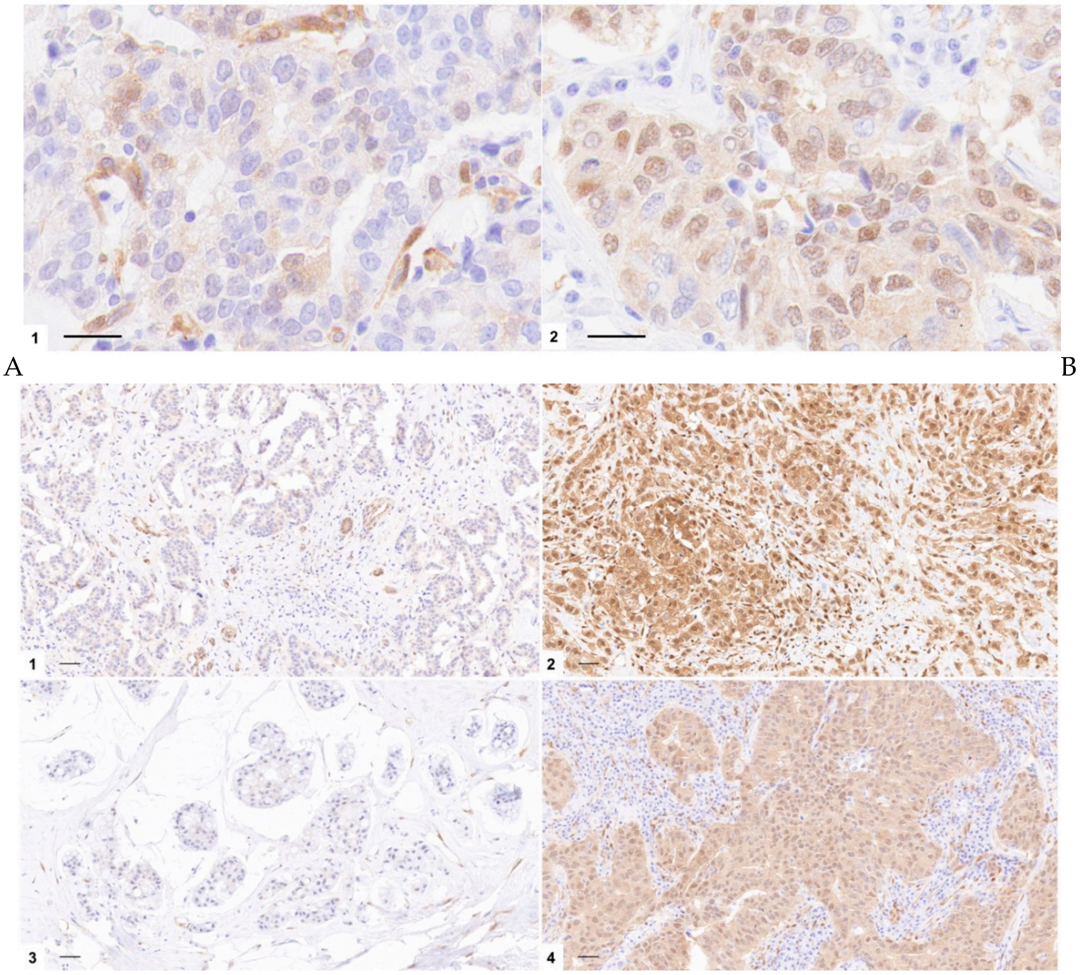

Figure S12. Representative IHC images of YAP1 staining from **A.** A paired sample pre-therapy (1) and post-therapy (2) with trastuzumab from a tumor of a patient who progressed to the treatment. **B.** Neoadjuvant samples from four different breast cancer patients. Bars: 50 μm. DAB stain; hematoxylin counterstain; microscopic magnification ×400.

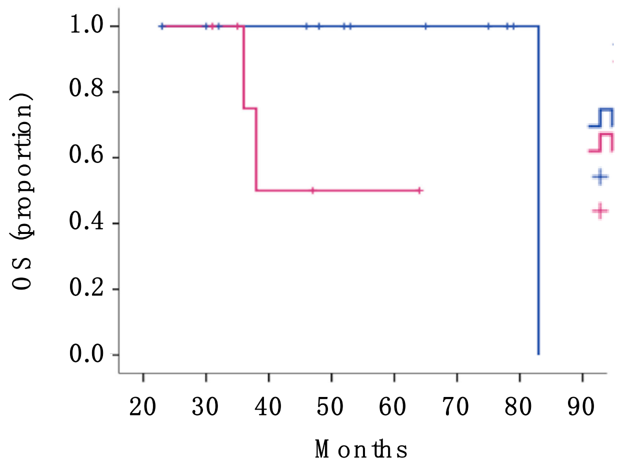

Figure S13. OS for pre- and post-treatment paired samples from metastatic breast cancer patients, stratified by upregulation or not of YAP1 expression levels ($p = 0.023$).

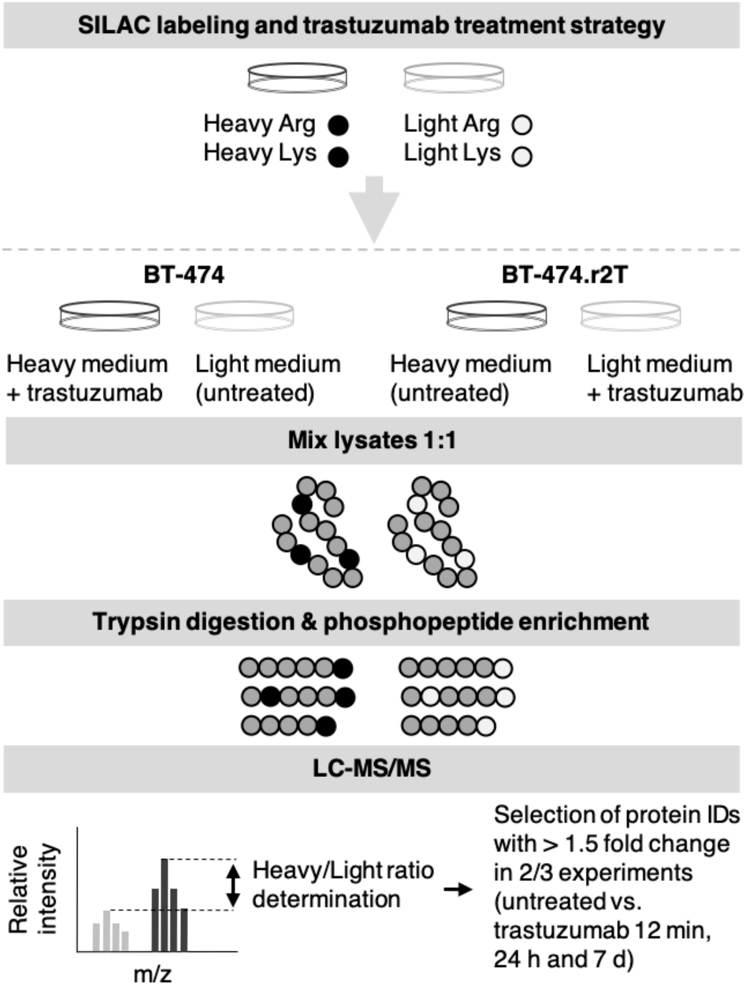

Figure S14. A. Experimental workflow for the SILAC experiment. BT-474 and BT-474.r2T cells were either untreated or treated with 15 µg/ml of trastuzumab for 12 min or 7 days and lysed. Lysates were digested and enriched for phosphopeptides by TiO_2 columns prior to mass spectrometry analysis. Protein identification was performed by MaxQuant and relative phosphorylation was determined by SILAC quantitation of phosphopeptides.

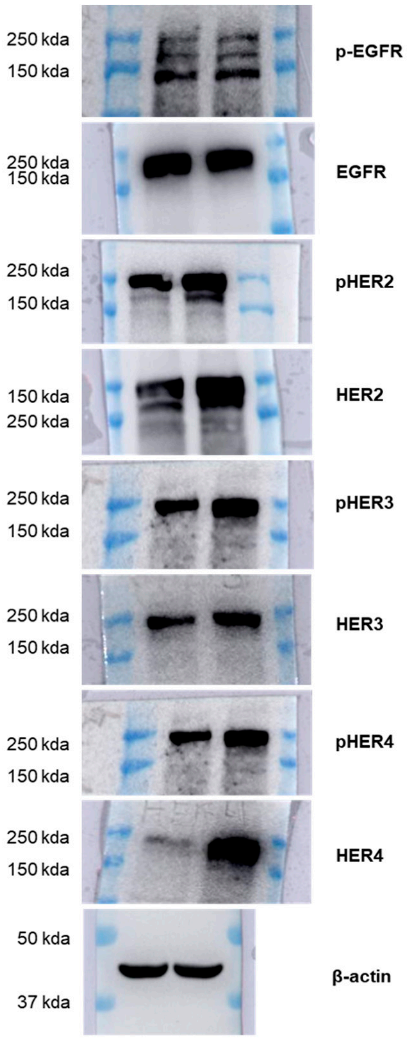

Protein	Raw data		Ratio		Relative	
	BT-474	BT-474.r2T	BT-474	BT-474.r2T	BT-474	BT-474.r2T
pEGFR	166298	176155	0,91	0,93	100,00	102,04
EGFR	211609	210196	1,16	1,11	100,00	95,69
p-HER2	70352	170345	0,39	0,90	100,00	233,25
HER2	78219	153037	0,43	0,81	100,00	188,48
p-HER3	177864	211527	0,98	1,12	100,00	114,56
HER3	122512	166497	0,67	0,88	100,00	130,92
p-HER4	124415	200999	0,68	1,06	100,00	155,63
HER4	59764	211967	0,33	1,12	100,00	341,67
β-actin	182139	189073	NP			

Figure 1A

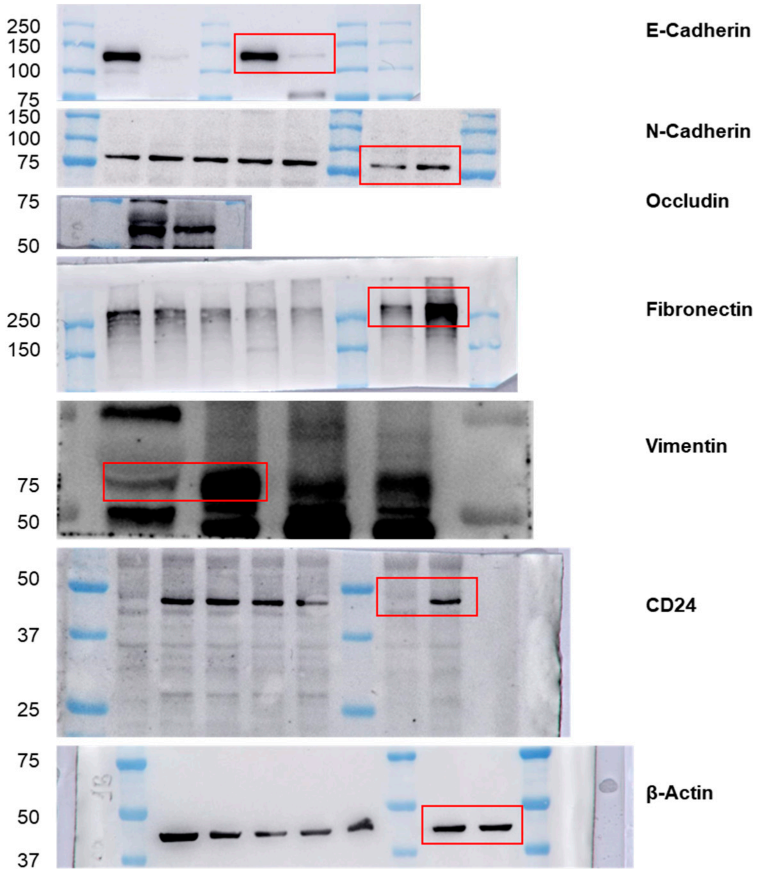

raw data

	E-Cadherin	Ocludin	N-Cadherin	Fibronectin	Vimentin	CD24	β-Actin
BT474	24289045	78984730	12384439	10041983	13997631	6074539	29846844
BT474.rT2	1587669	43901312	20689439	26010782	30548631	19903953	30178522

ratio

	E-Cadherin / β-actin	Ocludin / β-actin	N-Cadherin / β-actin	Fibronectin / β-actin	Vimentin / β-actin	CD24 / β-actin
BT474	0,81	2,65	0,41	0,34	0,47	0,20
BT474.rT2	0,05	1,45	0,69	0,86	1,01	0,66

normalization

	E-Cadherin	Ocludin	N-Cadherin	Fibronectin	Vimentin	CD24
BT474	1,00	1,00	1,00	1,00	1,00	1,00
BT474.rT2	0,06	0,55	1,65	2,56	2,16	3,24

Figure 1B

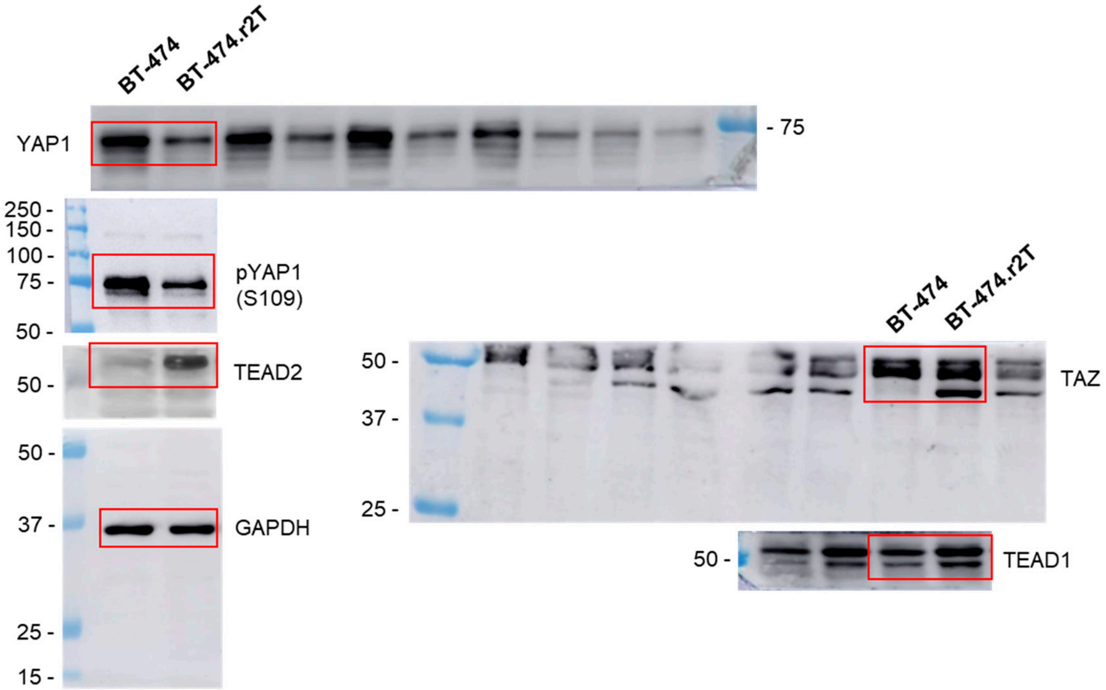

Protein	Raw data		Ratio		Relative	
	BT-474	BT-474.r2T	BT-474	BT-474.r2T	BT-474	BT-474.r2T
**YAP1**	211285	148269	1,34	1,01	100,00	75,18
**pYAP1 (S109)**	182536	121706	1,16	0,83	100,00	71,43
**TAZ**	136897	167048	0,87	1,14	100,00	130,74
**TEAD1**	155547	184760	1,17	1,06	100,00	90,20
**TEAD2**	28150602	46960584	178,55	319,12	100,00	178,73
**GAPDH**	157662	147157				

Figure 4B

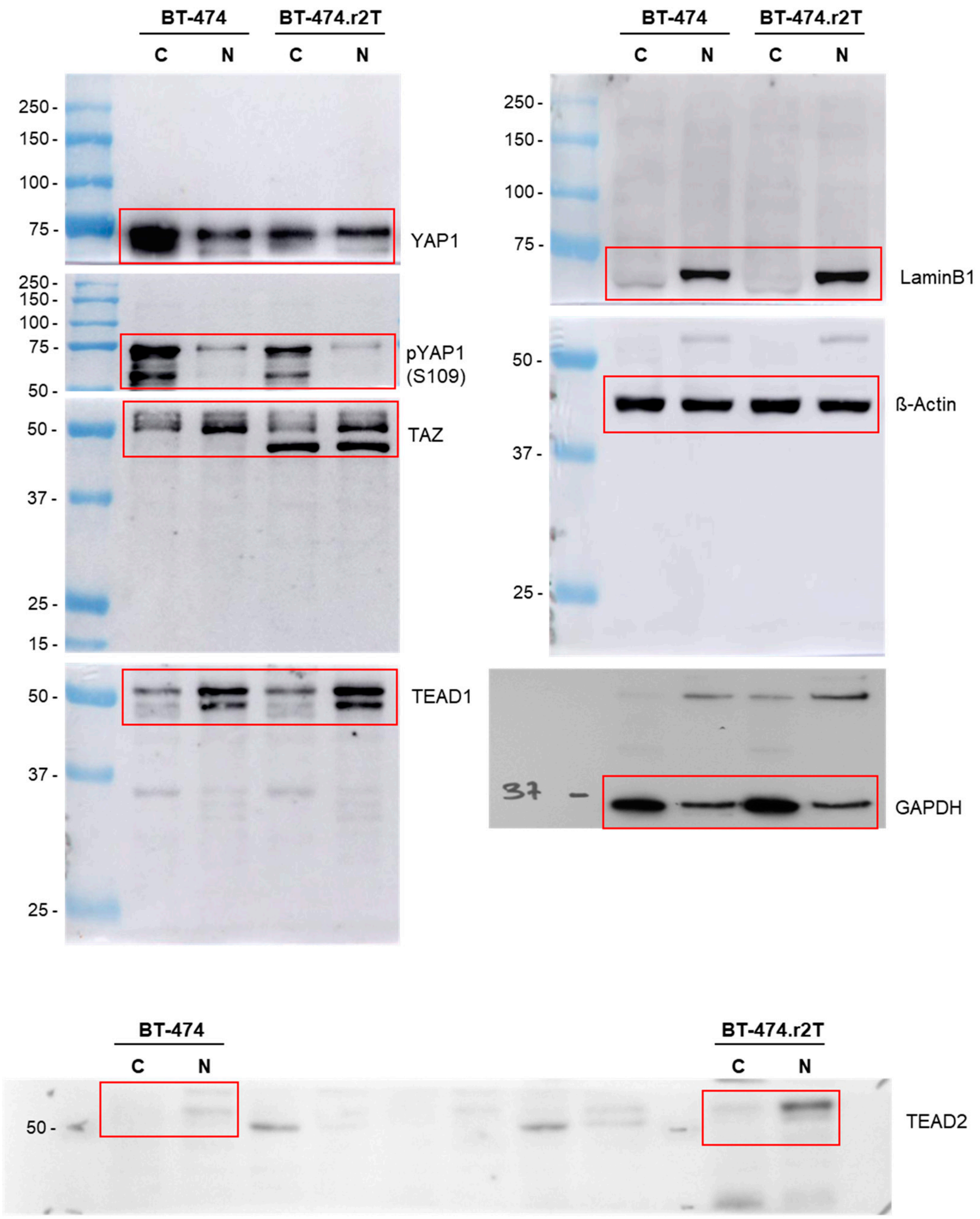
BT-474
BT-474.r2T
C
N
C
N
250 -
150 -
100 -
75 -
YAP1
50 -
pYAP1
(S109)
TAZ
37 -
25 -
15 -
TEAD1
LaminB1
ß-Actin
GAPDH
37 -
TEAD2

Figure 4C (I)

protein	intensity value	normalization1	normalization2	ratio lane#/lane1
ß-Actin	15946,15	1,00	1,00	1,00
	13982,00	0,88	1,00	1,00
	15478,42	0,97	1,00	1,00
	12669,30	0,79	1,00	1,00
YAP1	25460,82	1,60	1,60	1,00
	13686,77	0,98	1,12	0,70
	14974,72	0,97	1,00	0,62
	13999,02	1,10	1,39	0,87
pYAP1(S109)	24241,51	1,52	1,52	1,00
	6978,97	0,50	0,57	0,37
	21632,29	1,40	1,44	0,95
	4198,19	0,33	0,42	0,27
TAZ	9590,51	0,60	0,60	1,00
	16253,92	1,16	1,33	2,20
	17295,68	1,12	1,15	1,91
	23985,36	1,89	2,38	3,96
TEAD1	7197,54	0,45	0,45	1,00
	19430,41	1,39	1,58	3,51
	9115,27	0,59	0,61	1,34
	22687,63	1,79	2,25	4,99
TEAD2	810,02	0,05	0,05	1,00
	2102,05	0,15	0,17	3,38
	1996,04	0,13	0,13	2,62
	8378,37	0,66	0,83	16,39
laminB	3756,50	0,24	0,24	1,00
	11858,78	0,85	0,97	4,11
	2739,52	0,18	0,18	0,77
	14390,00	1,14	1,43	6,07
GAPDH	15526,15	0,97	0,97	1,00
	3964,00	0,28	0,32	0,33
	16457,36	1,06	1,10	1,13
	5567,64	0,44	0,55	0,57

Figure 4C (II)

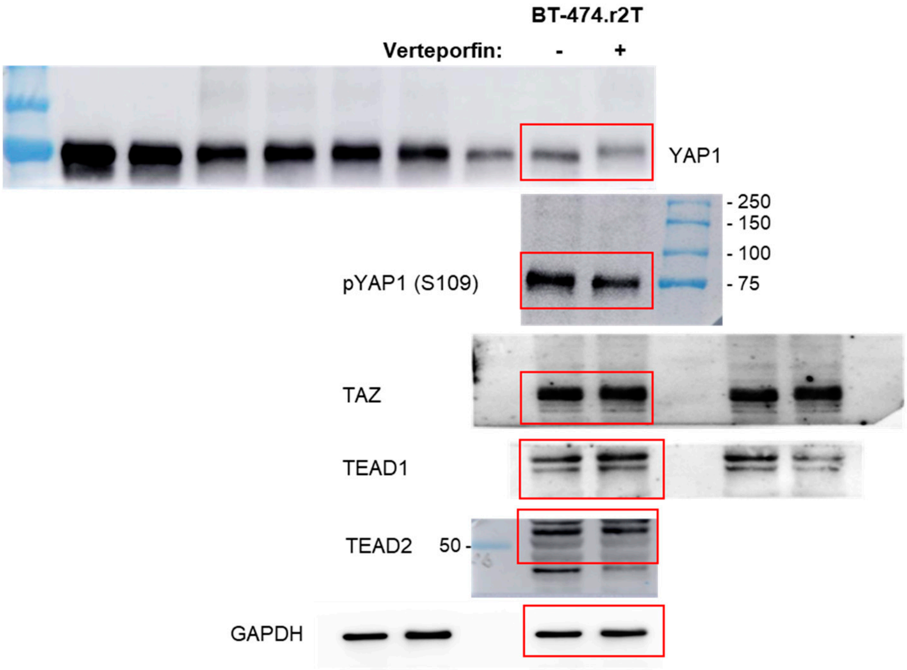

	raw data					
	**YAP1**	**p-YAP S109**	**TAZ**	**TEAD1**	**TEAD2**	**GAPDH**
**BT474**	4348477	38477421	35404057	31248936	51330602	20815205
**BT474.rT2**	3329770	27988744	38271844	41280472	45205693	23120933

	ratio				
	**YAP1/GAPDH**	**p-YAP S109/GAPDH**	**TAZ/GAPDH**	**TEAD1/GAPDH**	**TEAD2/GAPDH**
**BT474**	0,21	1,85	1,70	1,50	2,47
**BT474.rT2**	0,14	1,21	1,66	1,79	1,96

	normalization				
	**YAP1**	**p-YAP S109**	**TAZ**	**TEAD1**	**TEAD2**
**BT474**	1,00	1,00	1,00	1,00	1,00
**BT474.rT2**	0,69	0,65	0,97	1,19	0,79

Figure 6B

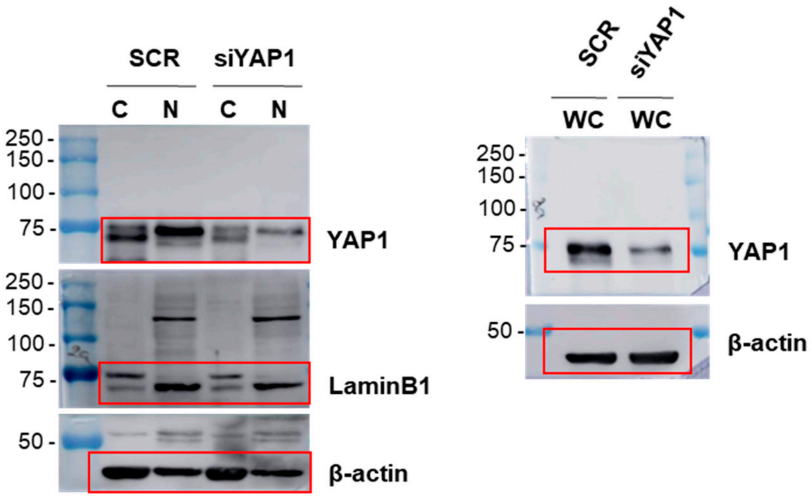

	**raw data**					
	**siYAP1 fractionated**				**siYAP1 WC**	
	**YAP1**	**LaminB1**	**β-actin**		**YAP1**	**β-actin**
**SCR C**	21068652	8094004	22367865	**SCR**	32832915	50789329
**SCR N**	25560752	14298853	10418116	**siYAP1**	12192702	49996551
**siYAP1 C**	11569731	6235418	23511602			
**siYAP1 N**	8905853	9513681	11223785			

	**ratio**				
	**siYAP1 fractionated**			**siYAP1 WC**	
	**YAP1 / LaminB1**	**YAP1 / β-actin**		**YAP1 / β-actin**	
**SCR C**	2,60	0,94	**SCR**	0,65	
**SCR N**	1,79	2,45	**siYAP1**	0,24	
**siYAP1 C**	1,86	0,49			
**siYAP1 N**	0,94	0,79			

	**normalization**		
	**siYAP1 fractionated**		**siYAP1 WC**
	**LaminB1**		**YAP1**
**SCR N**	1,00	**SCR**	1,00
**siYAP1 N**	0,52	**siYAP1**	0,38
	**β-actin**		
**SCR C**	1,00		
**siYAP1 C**	0,52		

Supplementary Figure S5(I)

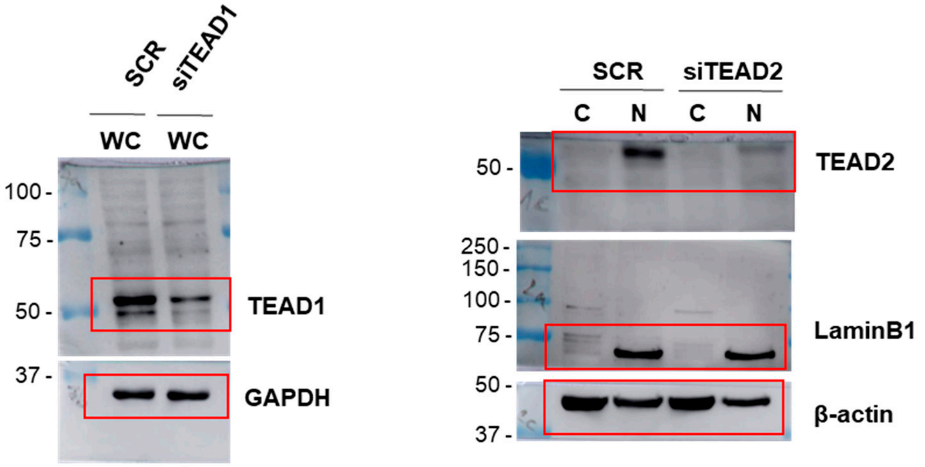

raw data

	siTEAD2 fractionated				siTEAD1 WC	
	TEAD2	LaminB1	β-actin		TEAD1	β-actin
SCR C	4229986	4688986	25560095	SCR	37021744	22324317
SCR N	22558095	30154756	15138317	siTEAD1	16838744	22805903
siTEAD2 C	3077983	1905861	22927560			
siTEAD2 N	9330844	20404404	14134317			

ratio

	siTEAD2 fractionated			siTEAD1 WC
	TEAD2 / LaminB1	TEAD2 / β-actin		TEAD1 / β-actin
SCR C	0,90	0,17	SCR	1,66
SCR N	0,75	1,49	siTEAD1	0,74
siTEAD2 C	1,62	0,13		
siTEAD2 N	0,46	0,66		

normalization

	siTEAD2 fractionated		siTEAD1 WC
	LaminB1		TEAD1
SCR N	1,00	SCR	1,00
siTEAD2 N	0,61	siTEAD1	0,45
	β-actin		
SCR C	1,00		
siTEAD2 C	1,23		

Supplementary Figure S5 (II)

Figure S15. Original WB images and densitometric analysis for all the WB figures presented in this work.

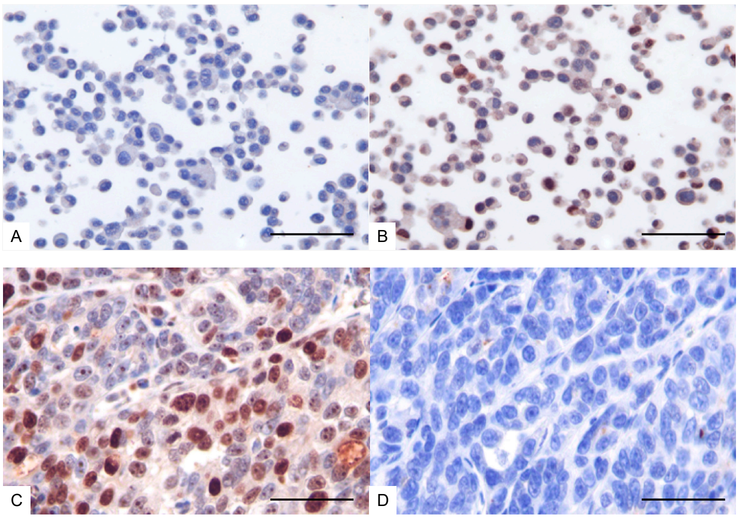

Figure S16. Positive control on BT-474 cells. Formalin-fixed and paraffin-embedded pellets were obtained from parental, trastuzumab-sensitive BT-474 cell line (**A**) and resistant (**B**) BT-474.r2T cells. IHC assay for YAP1 expression was performed as described. Nuclear and faint cytoplasmic YAP1 was detected in **B**, whereas complete absence of expression was seen in **A**. Negative control in selected breast carcinoma. Sections from same specimen were incubated with YAP1 antibody (**C**) and with non-immunized immunoglobulin instead primary antibody (**D**) to demonstrate the specificity of staining method. Bars: 50 µm. DAB stain; hematoxylin counterstain; microscopic magnification x400.

Table S1. Summary table of the proteins identified in the SILAC-based approach to identify differential regulation of the trastuzumab-resistant BT-474.r2T cell line.

Identification Level	12 min		24 h		7 d		$\overline{X}$	
	N	%	N	%	N	%	N	%
Protein groups	1233		1978		1826		1679.00	
Peptides	71786		97575		99872		89744.33	
Phosphosites	1261		2527		2231		2006.33	
Class I phosphosites (>0.75)	939	74.46	1869	73.96	1665	74.63	1491.00	74.35

Table S2. GSEA of the differentially regulated genes in BT-474.r2T cells as compared to BT-474 (Molecular Signatures Database, Oncogenic Signature). Only top significant gene sets with FDR *q*-val < 0.005 are shown. **A.** Analysis for BT-474.r2T cells at baseline conditions. **B.** When BT-474.r2T cells were treated with trastuzumab, a couple of extra gene sets were revealed in the analysis.

A

Phenotype	Gene Set	Description	NES	FDR q-value
Basal condition	ERB2_UP (V1_DN)	Correlated with breast cancer cells ERBB2-positive	2.8128	0.0000
	LTE2_UP (V1_DN)	Long-term adapted for estrogen-independent growth	2.6767	0.0000
	MEK_UP (V1_DN)	Expression of MAP2K1 gene	2.4962	0.0000
	EGFR_UP (V1_DN)	Correlated with breast cancer cells EGFR-positive	2.0859	0.0000

B

Phenotype	Gene Set	Description	NES	FDR q-value
Trastuzumab treatment	ERB2_UP (V1_DN)	Correlated with breast cancer cells ERBB2-positive	2.6952	0.0000
	LTE2_UP (V1_DN)	Long-term adapted for estrogen-independent growth	2.4201	0.0000
	CSR_LATE_UP (V1_UP)	Correlated to fibroblast serum response	2.3719	0.0000
	RB_P107_DN (V1_UP)	Correlated to RB1 and RBL1 specific knockouts	2.3278	0.0000
	MEK_UP (V1_DN)	Expression of MAP2K1 gene	2.2924	0.0000

Table S3. Overlapping of two out of three time data sets (BH corrected *p*-value ≤ 0.05).

# Accession	GO biological process	Observed gene count	Corrected p-value	Matching genes and proteins in network
GO:0009615	response to virus	13	1.97E-07	BAD, CCL5, CXCL10, DDX21, HERC5, IFI44, IFI44L, IFIT1, IFIT2, IFIT3, IL28A, IL29, OAS2
GO:0051607	defense response to virus	9	0.00015	CXCL10, HERC5, IFI44L, IFIT1, IFIT2, IFIT3, IL28A, IL29, OAS2
GO:0009607	response to biotic stimulus	15	0.000725	ASS1, BAD, CCL5, CXCL11, DDX21, HERC5, IFI44, IFI44L, IFIT1, IFIT2, IFIT3, IL28A, IL29, OAS2, TP53
GO:0051707	response to other organism	14	0.00163	ASS1, BAD, CCL5, CXCL11, DDX21, HERC5, IFI44, IFI44L, IFIT1, IFIT2, IFIT3, IL28A, IL29, OAS2
GO:0006955	immune response	19	0.00188	ASS1, BAD, BPIFB1, CCL5, CD276, CDK1, CXCL10, CXCL11, ERBB2, HERC5, IFI44L, IFIT1, IFIT2, IFIT3, IL28A, IL29, IRS1, OAS2, TP53
GO:0009967	positive regulation of signal transduction	19	0.00188	BAD, BCLAF1, CCL5, CDK1, CXCL10, CXCL11, DBNL, ERBB2, FLNA, IL29, IRS1, LASP1, MED1, PCM1, PEA15, PLEKHF1, PXN, TP53, YAP1
GO:0023056	positive regulation of signaling	19	0.00649	BCLAF1, CCL5, CDK1, CXCL10, CXCL11, DBNL, DKK1, ERBB2, FLNA, IL29, IRS1, LASP1, MED1, PCM1, PEA15, PLEKHF1, PXN, TP53, YAP1
GO:0002376	immune system process	22	0.0126	ASS1, BAD, BPIFB1, CCL5, CD276, CDK1, CUL4A, CXCL11, DBNL, ERBB2, HERC5, IFI44L, IFIT1, IFIT2, IFIT3, IKZF3, IL28A, IL29, IRS1, OAS2, PCM1, TP53
GO:0002252	immune effector process	10	0.0145	CXCL10, HERC5, IFI44L, IFIT1, IFIT2, IFIT3, IL28A, IL29, OAS2, TP53
GO:0072676	lymphocyte migration	4	0.0145	CCL5, CXCL10, CXCL11, PCM1
GO:0010647	positive regulation of cell communication	19	0.0149	BCLAF1, CCL5, CDK1, CXCL10, CXCL11, DBNL, DKK1, ERBB2, FLNA, IL29, IRS1, LASP1, MED1, PCM1, PEA15, PLEKHF1, PXN, TP53, YAP1
GO:0010941	regulation of cell death	18	0.0168	BAD, BCLAF1, CCL5, CDK1, CTTN, FLNA, HEY2, IFIT2, IFIT3, IKZF3, MED1, PCM1, PIP, PLEKHF1, SET, TEAD2, TP53, YAP1
GO:0045595	regulation of cell differentiation	18	0.0168	ARHGAP35, CCL5, CD276, CDK1, CTTN, CUL4A, CXCL10, DBN1, DKK1, ERBB2, FLNA, HEY2, IKZF3, IL29, MED1, PCM1, TEAD2, TP53
GO:0060548	negative regulation of cell death	14	0.0168	BAD, CCL5, CDK1, CTTN, FLNA, HEY2, IFIT3, MED1, PEA15, PIP, SET, TEAD2, TP53, YAP1
GO:0045087	innate immune response	14	0.0191	ASS1, BAD, BPIFB1, CCL5, CDK1, ERBB2, HERC5, IFIT1, IFIT2, IFIT3, IL28A, IL29, IRS1, OAS2

GO:1902533	positive regulation of intracellular signal transduction	13	0.0209	BAD, BCLAF1, CCL5, CDK1, CXCL10, CXCL11, DBNL, ERBB2, FLNA, IL29, PLEKHF1, PXN, TP53
GO:0019221	cytokine-mediated signaling pathway	9	0.0219	BAD, CCL5, CXCL10, CXCL11, HERC5, IFIT1, IFIT2, IFIT3, OAS2
GO:0006333	chromatin assembly or disassembly	6	0.0228	H1FX, HIRIP3, MCM2, SET, SMARCC1, TP53
GO:0043066	negative regulation of apoptotic process	13	0.023	BAD, CCL5, CDK1, CTTN, FLNA, HEY2, IFIT3, MED1, PEA15, PIP, SET, TP53, YAP1
GO:0060429	epithelium development	14	0.023	ARHGAP35, CDK1, DKK1, EMP1, HEY2, KRT4, PCM1, PXN, SERPINB5, TACSTD2, TEAD2, TP53, UPK1A, YAP1
GO:0071822	protein complex subunit organization	17	0.0273	AHNAK, BAD, CCL5, CTTN, DBN1, DBNL, FLNA, FMOD, H1FX, MAP4, MCM2, PCM1, PXN, SET, SMARCC1, TP53, UPK1A
GO:0051097	negative regulation of helicase activity	2	0.0313	IFIT1, TP53
GO:0060284	regulation of cell development	12	0.0313	ARHGAP35, BAD, CCL5, CDK1, CTTN, DBN1, FLNA, HEY2, MED1, PCM1, TACSTD2, TP53
GO:0048247	lymphocyte chemotaxis	3	0.0321	CCL5, CXCL10, CXCL11
GO:0048522	positive regulation of cellular process	33	0.0321	ARHGAP35, ASS1, BAD, BCLAF1, CCL5, CDK1, CTTN, CUL4A, CXCL10, CXCL11, DBNL, DKK1, ERBB2, FLNA, HEY2, HUWE1, IFIT1, IFIT2, IKZF3, IL29, IRS1, LASP1, MARCKSL1, PCM1, PEA15, PLEKHF1, PXN, SPTBN1, TACSTD2, TEAD2, TP53, VAPB, YAP1
GO:0043933	macromolecular complex subunit organization	21	0.0325	AHNAK, BAD, C17orf49, CCL5, CTTN, DBN1, DBNL, FLNA, FMOD, H1FX, HEY2, HIRIP3, HUWE1, MAP4, MCM2, PCM1, PXN, SET, SMARCC1, TP53, UPK1A
GO:0048518	positive regulation of biological process	36	0.0344	ARFGEF2, ASS1, BAD, BCLAF1, CCL5, CDK1, CTTN, CUL4A, CXCL10, CXCL11, DBNL, DKK1, ERBB2, FLNA, HEY2, HUWE1, IFIT1, IFIT2, IKZF3, IL28A, IL29, IRS1, LASP1, MARCKSL1, PCM1, PEA15, PIP, PLEKHF1, PXN, SPTBN1, TACSTD2, TBC1D9B, TEAD2, TP53, VAPB, YAP1
GO:0045596	negative regulation of cell differentiation	11	0.0357	CUL4A, CXCL10, DKK1, ERBB2, HEY2, IL29, MED1, PCM1, TACSTD2, TP53, YAP1

GO:0032388	positive regulation of intracellular transport	8	0.0384	BAD, CXCL10, CXCL11, FLNA, HUWE1, MED1, PCM1, TP53
GO:0071840	cellular component organization or biogenesis	35	0.0384	AHNAK, ARFGEF2, ARHGAP35, C17orf49, C19orf21, CCL5, CDK1, COG3, DBN1, DDX21, DKK1, EMP1, FLNA, FMOD, H1FX, HEY2, HIRIP3, HUWE1, IFIT2, KRT4, MAP4, MCM2, MED1, NEK9, PCM1, PLEC, PLEKHF1, SERPINB5, SET, SMARCC1, SPTBN1, TNKS1BP1, UPK1A, VAPB, YAP1
GO:2001244	positive regulation of intrinsic apoptotic signaling pathway	4	0.0384	BAD, BCLAF1, PLEKHF1, TP53
GO:0042981	regulation of apoptotic process	16	0.0444	BCLAF1, CCL5, CDK1, CTTN, FLNA, HEY2, IFIT2, IFIT3, IKZF3, MED1, PCM1, PIP, PLEKHF1, SET, TP53, YAP1
GO:0006356	regulation of transcription from RNA polymerase I promoter	3	0.0452	ERBB2, FLNA, MED1
GO:0006461	protein complex assembly	13	0.0461	AHNAK, BAD, CCL5, CTTN, DBNL, FLNA, FMOD, H1FX, MCM2, PXN, SET, TP53, UPK1A
GO:0007166	cell surface receptor signaling pathway	20	0.0461	CCL5, CDK1, CXCL10, CXCL11, DKK1, ERBB2, FMOD, HERC5, HEY2, IFIT1, IFIT2, IFIT3, IRS1, OAS2, P2RY10, PCM1, PXN, SMARCC1, SPTBN1, TACSTD2
GO:0016043	cellular component organization	34	0.0461	AHNAK, ARFGEF2, ARHGAP35, C17orf49, C19orf21, CCL5, CDK1, COG3, DBN1, DKK1, EMP1, FLNA, FMOD, H1FX, HEY2, HIRIP3, HUWE1, IFIT2, KRT4, MAP4, MCM2, MED1, NEK9, PCM1, PLEC, PLEKHF1, SERPINB5, SET, SMARCC1, SPTBN1, TNKS1BP1, UPK1A, VAPB, YAP1
GO:0050678	regulation of epithelial cell proliferation	7	0.0461	BAD, CCL5, ERBB2, KRT4, SERPINB5, TACSTD2, YAP1
GO:0050767	regulation of neurogenesis	10	0.0461	ARHGAP35, CCL5, CDK1, CTTN, DBN1, HEY2, MED1, PCM1, TP53, YAP1
GO:0070271	protein complex biogenesis	13	0.0461	AHNAK, BAD, CCL5, CTTN, DBNL, FLNA, FMOD, H1FX, MCM2, PXN, SET, TP53, UPK1A
GO:0071396	cellular response to lipid	8	0.0461	ASS1, BAD, CCL5, CXCL10, MED1, PCM1, TEAD2, YAP1
GO:2001235	positive regulation of apoptotic signaling pathway	6	0.0461	BAD, BCLAF1, PCM1, PEA15, PLEKHF1, TP53
GO:0048523	negative regulation of cellular process	30	0.0462	ASS1, BAD, BCLAF1, BPIFB1, CCL5, CD276, CTTN, CXCL10, DKK1, ERBB2, FLNA, HEY2, IFIT1, IFIT3, IL29, IRS1, KRT4, MED1, PCM1, PEA15, PIP, SERPINB5, SET, SMARCC1, SPTBN1, TACSTD2, TCEAL1, TEAD2, TP53, VAPB
GO:0048584	positive regulation of response to stimulus	19	0.0475	BCLAF1, CCL5, CDK1, CXCL10, CXCL11, DBNL, ERBB2, FLNA, IL28A, IL29, IRS1, LASP1, MED1, PCM1, PEA15, PLEKHF1, PXN, TP53, YAP1

Table S4. Correlation between YAP1 and TAZ abundance levels, as determined by IHC, in the cohort of 58 samples.

Molecular, clinical and pathological indicator		Number of samples (n=58)	%	Low-level TAZ (n=44)		High-level TAZ (n=14)		p (from χ^2 test)
				N	%	N	%	
TAZ expression		58		44	75.9	14	24.1	
YAP1 expression								< 0.001
	Low level	46	79.3	42	91.3	4	8.7	
	High level	12	20.7	2	16.7	10	83.3	
Age (years) (median, range)		51 (32-86)						
Hormonal status								0.22
	Premenopausal	29	50	24	82.8	5	17.2	
	Postmenopausal	29	50	20	69	9	31	
Type of breast cancer*								0.569
	NOS	57	98.3	43	75.4	14	24.6	
	ILC	1	1.7	1	100	0	0	
Histological grade								0.663
	G1	1	1.7	100	0	0	1	
	G2	37	63.8	78.4	8	21.6	29	
	G3	20	34.5	70	6	30	14	
ER status								0.766
	Negative	31	53.4	24	77.4	7	22.6	
	Positive	27	46.6	20	74.1	7	25.9	
PR status								0.702
	Negative	39	67.2	29	74.4	10	25.6	
	Positive	19	32.8	15	78.9	4	21.1	
Proliferation (Ki-67)								0.634
	<20%	10	17.2	7	70	3	30	
	≥20%	48	82.8	37	77.1	11	22.9	
Progression								< 0.001
	Long progression	49	84.5	43	97.7	6	42.9	
	Early progression	9	15.5	1	2.3	8	57.1	

* Type of breast cancer: NOS: invasive ductal carcinoma, not otherwise specified; ILC: invasive lobular carcinoma.

Table S5. Multivariate analysis of YAP1 expression and clinical and pathological indicators. Hormonal status and YAP1 act as prognostic factors, independently of all the other clinical and pathological indicators. HR: hazard ratio; CI: confidence interval.

Indicator	HR	95% CI		*p*
		Lower	Upper	
Hormonal status	2.880	1.005	8.251	<0.05
ER status	0.707	0.265	1.888	0.489
YAP1 expression	4.789	1.776	12.911	<0.01

Table S6. List of primers. The forward (FW) and reverse (RV) sequences of each primer used in this study is provided.

qPCR Primer	Sequence (5'-3')
AREG-FW	GTTATTACAGTCCAGCTTAGAAG
AREG-RV	CATGTACATTTCCATTCTCTTG
ATP5E-FW	CCGGCGTCTTGGCGATTC
ATP5E-RV	GATCTGGGAGTATCGGATG
CTGF-FW	GTTACCAATGACAACGCCTC
CTGF-RV	GAGTACGGATGCACTTTTTG
CYR61-FW	GTTACCAATGACAACCCTGAG
CYR61-RV	GGGATTTCTTGGTCTTGCTG
TAZ-FW	CAAGTCGGCTGTGGAGATG
TAZ-RV	CTGGAGGTGGTTGTGGAGC
TEAD1-FW	GAAAACATGGAAAGGATGAGTG
TEAD1-RV	GGCTATCAATTCATTCCTACC
TEAD2-FW	GAGCGATACATGATGAACAG
TEAD2-RV	GGAGACCTCGAAGACATAGG
TEAD3-FW	GATTGCACGCTATATTAAACTGAG
TEAD3-RV	CTTGATGCCAACCTGGTACTC
TEAD4-FW	CTTTCTCTCAGCAAACCTATG
TEAD4-RV	GAGAACTCCAACATCCAGAG
VEGFA-FW	TGTCTTGGGTGCATTGGAG
VEGFA-RV	GATTCTGCCCTCCTCCTTCTG
YAP1-FW	GGCTGAAACAGCAAGAACTG
YAP1-RV	GAAGACACTGGATTTTGAGTC

© 2020 by the authors. Licensee MDPI, Basel, Switzerland. This article is an open access article distributed under the terms and conditions of the Creative Commons Attribution (CC BY) license (http://creativecommons.org/licenses/by/4.0/).
